# Construction and Validation of an Immune-Related Gene Prognostic Index for Esophageal Squamous Cell Carcinoma

**DOI:** 10.1155/2021/7430315

**Published:** 2021-10-21

**Authors:** Qinghua Ji, Yingying Cai, Sachin Mulmi Shrestha, Duo Shen, Wei Zhao, Ruihua Shi

**Affiliations:** ^1^Medical School, Southeast University, Nanjing, China; ^2^Department of Gastroenterology, Zhongda Hospital, Southeast University, Nanjing, China; ^3^The Second Hospital of Nanjing, Southeast University, Nanjing, China

## Abstract

Immune checkpoint inhibitor (ICI) therapy may benefit patients with advanced esophageal squamous cell carcinoma (ESCC); however, novel biomarkers are needed to help predict the response of patients to treatment. Differentially expressed immune-related genes within The Cancer Genome Atlas ESCC dataset were selected using the weighted gene coexpression network and lasso Cox regression analyses. Based on these data, an immune-related gene prognostic index (IRGPI) was constructed. The molecular characteristics of the different IRGPI subgroups were assessed using mutation information and gene set enrichment analysis. Differences in immune cell infiltration and the response to ICI therapy and other drugs were also analyzed. Additionally, tumor and adjacent control tissues were collected from six patients with ESCC and the expression of these genes was verified using real-time quantitative polymerase chain reaction. IRGPI was designed based on *CLDN1*, *HCAR3*, *FNBP1L*, and *BRCA2*, the expression of which was confirmed in ESCC samples. The prognosis of patients in the high-IRGPI group was poor, as verified using publicly available expression data. *KMT2D* mutations were more common in the high-IRGPI group. Enrichment analysis revealed an active immune response, and immune infiltration assessment showed that the high-IRGPI group had an increased infiltration degree of CD8 T cells, which contributed to the improved response to ICI treatment. Collectively, these data demonstrate that IRGPI is a robust biomarker for predicting the prognosis and response to therapy of patients with ESCC.

## 1. Introduction

Esophageal carcinoma is one of the leading causes of cancer-related death worldwide [1]. The main types of esophageal carcinoma are squamous cell carcinoma (ESCC) and adenocarcinoma, which exhibit different pathomorphological, epidemiological, and molecular characteristics [2]. Moreover, ESCC accounts for approximately 90% of the morbidity and mortality of all esophageal cancers [3]. The promotion of large-scale population screening has effectively improved the survival rate of patients with ESCC; nonetheless, a considerable number of patients with ESCC are in an advanced stage at diagnoses [4]. Only 27% of patients with ESCC benefit from traditional chemotherapy and radiotherapy [5, 6]. Therefore, novel methods to predict the prognosis of patients with ESCC and their corresponding treatment are urgently needed.

An increasing number of researchers have recently focused on immune checkpoint inhibitor (ICI) therapy, which prevents tumor cell immune escape and induces an immune response by inhibiting immune checkpoints, such as programmed death-1 (PD-1), programmed death-ligand 1 (PD-L1), and CTL-associated protein-4 (CTLA-4) pathways [7–9]. Phase III ICI clinical trials, including those on nivolumab and pembrolizumab for ESCC, showed that ICIs can significantly improve the patient survival rate and reduce the incidence of treatment-related adverse events compared with traditional chemotherapy drugs [7–9]. Most patients achieve cancer control upon ICI therapy, although some develop recurrence or drug resistance [10]. Moreover, approximately 10% patients treated with ICIs may have unconventional response patterns (pseudoprogression), which challenges the evaluation of treatment efficacy [11]. Thus, methods for evaluating whether patients are suitable for ICI therapy and assessing ICI therapy efficacy are urgently needed.

The response of patients to ICI therapy is mainly affected by tumor cell-intrinsic factors (such as tumor mutational burden and microsatellite instability) and the tumor microenvironment [12, 13]. Hence, evaluation of the immune status of the tumor microenvironment by immune gene signatures may effectively help predict the benefits of ICIs [14]. The expression level of immune-related genes can predict the response of patients to ipilimumab treatment [15]. Additionally, recent studies showed that immune- or tumor microenvironment-related gene expression scores can predict the survival and response to immunotherapy in hepatocellular carcinoma and lung cancer [16, 17]. Therefore, it is helpful to evaluate the immune microenvironment, prognosis, and response to immunotherapy by examining gene expression in patients before treatment.

**Figure 1:**
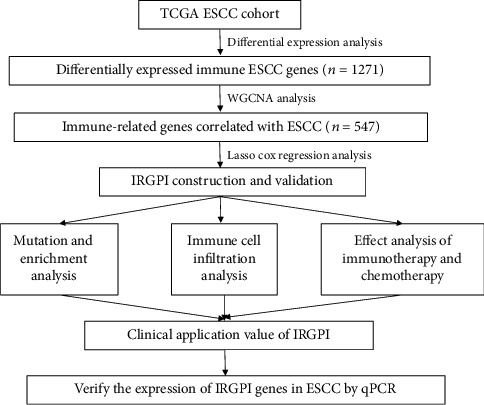
Experimental strategy for IRGPI development in ESCC.

In this study, we used immune gene signatures to develop prognostic and ICI therapy indicators for patients with ESCC. We also performed weighted gene coexpression network analysis (WGCNA) and lasso regression analysis to construct an immune-related gene prognostic index (IRGPI). The molecular and immune characteristics of the IRGPI subgroups were evaluated, and the potential of IRGPI for assessing immunotherapy efficacy in patients with ESCC was determined. The study design is shown in Figure 1.

## 2. Materials and Methods

### 2.1. Collection of Patient Information and Databases

ESCC transcriptome data, clinical information, and gene mutation data were downloaded from The Cancer Genome Atlas (TCGA) database, which included 81 tumor and 11 adjacent noncancerous samples. Transcriptome data and clinical information of the validation cohort GSE53625 with 179 ESCC tumor samples and 179 adjacent normal tissues were downloaded from the NCBI Gene Expression Omnibus database [18]. Immune-related gene lists were downloaded from InnateDB (https://www.innatedb.com), ImmPort, and IRIS [19–21].

### 2.2. Identification of Immune Genes Correlated with Prognosis

Differentially expressed ESCC genes were identified using the R package limma (version 3.44) based on TCGA transcriptome data, with a false discovery rate < 0.05 and fold change > 1.5 [22]. The differentially expressed immune ESCC genes were determined after the intersection of immune-related gene sets (InnateDB) with differentially expressed genes in ESCC. Gene Ontology (GO) analysis of these genes was performed using the R package clusterProfiler (version 3.17.5) [23].

The WGCNA (version 1.46) method was used to identify hub genes that were significantly associated with ESCC [24]. We used the scale-free topology criterion to determine the soft threshold of *β* = 7. Under this selection, the scale-free topology fitting index *R*^2^ > 0.85. Based on the gene expression matrix, the similarity of gene expression was calculated to obtain the adjacency matrix, which was then transformed into a topological overlap matrix. Genes were grouped by hierarchical clustering and then divided into different expression modules according to the coexpression pattern. We then calculated the correlation between these gene modules and ESCC occurrence. Modules with an absolute value of the correlation coefficient > 0.7 were selected for further analysis.

Univariate prognostic analysis was performed for genes in the selected modules with the R package survival (version 3.2). The R package glmnet (version 4.0) was used for lasso analysis of survival-related genes in univariate analysis [25]. The immune-related genes selected using lasso analysis were used to construct the prognosis index model.

### 2.3. IRGPI Construction and Reliability Evaluation

For immune-related genes included in the IRGPI, the R package survminer (version 0.4.8) was used to determine the optimal cutoff expression value for prognosis and the logrank test and Kaplan–Meier survival curve were used to determine its relationship with overall survival [26, 27]. Based on the immune-related genes selected, multivariate regression analysis using the R package rms (version 6.1) was performed to construct the IRGPI model. The IRGPI score of each sample was assessed by calculating the sum of each immune-related gene expression value multiplied by its corresponding regression coefficient based on the Cox proportional hazard model. The risk score plot of IRGPI was obtained using the R package ggrisk (version 1.2). The survivalROC (version 1.0.3) package was used to test the diagnostic significance of IRGPI in patients with ESCC in different years. TCGA patient data were divided into two groups according to the IRGPI median value. The Kaplan–Meier survival curve and logrank test were used to evaluate the prognosis value of IRGPI in TCGA ESCC cohort, which was further confirmed in the GSE53625 dataset.

### 2.4. Identification of Mutation and Enrichment Characteristics in Different IRGPI Subgroups

Based on the median value of IRGPI (0.13), TCGA ESCC patients were divided into high- and low-IRGPI groups. The R package limma (version 3.44) was used to analyze the differentially expressed genes between groups. The R package maftools (version 2.6.05) was used to summarize and visualize mutation information between subgroups [28]. The R package Pi (version 2.2.1) was used for gene set enrichment analysis (GSEA) between IRGPI subgroups based on the MSigDB hallmark gene set [29].

### 2.5. Evaluation of Immune Cell Infiltration

The ssGSEA function of the R package GSVA (version 1.36) was used to calculate the enrichment score of 28 types of immune cells for each sample [30]. The R packages ggpubr (version 0.4) and ggplot2 (version 3.3.0) were used to compare and visualize the immune cell enrichment score between groups. The R package ComplexHeatmap (version 2.7.9) was used to display the landscape map of the relationship between the infiltration of immune cells and clinical information of the samples [31].

### 2.6. Comparison of Immunotherapy Effectiveness and Chemotherapeutic Response

The SubMap module in GenePattern (https://cloud.genepattern.org/gp) was applied to predict the response effectiveness of the IRGPI subgroups to immunotherapy [32]. Expression data from patients with melanoma who responded to CTLA-4 and PD-1 therapy were compared with those of TCGA ESCC samples upon Bonferroni correction [33].

According to the Genomics of Drug Sensitivity in Cancer (GDSC) database (https://www.cancerrxgene.org/), we predicted the different reactions of the two IRGPI subgroups to chemotherapy. Based on the gene expression of samples and GDSC training set, the half-maximal inhibitory concentration (IC_50_) of each chemotherapy was evaluated by ridge regression using the R package pRRophetic (version 0.5) [34]. Tenfold crossvalidation was used to ensure prediction accuracy.

### 2.7. ESCC Tissue Collection

After obtaining ethics approval (no. 2019ZDSYLL023-Y01) from Zhongda Hospital, ESCC and adjacent-control tissues were collected from six patients with ESCC. The adjacent tissues were collected from the esophageal tissues that were more than 2 cm and less than 5 cm away from the ESCC tissues. Fresh ESCC and adjacent normal tissues were collected during surgery. After rapid freezing in liquid nitrogen, all tissue samples were stored at −80°C. The obtained tumor tissues were pathologically verified.

### 2.8. RNA Extraction and Quantitative Polymerase Chain Reaction (qPCR)

The FastPure Cell/Tissue Total RNA Isolation Kit (Vazyme, Nanjing, China) was used according to the manufacturer's instructions to extract total RNA from patient tissue samples. cDNA was synthesized using HiScript III RT SuperMix for qPCR (Vazyme). Using ChamQ SYBR qPCR Master Mix (Vazyme), qPCR was performed. The gDNA filter columns in the RNA extraction kit and subsequent gDNA wiper mix before reverse transcription ensured that there was little or no gDNA residue in the qPCR system. Relative gene expression was calculated using the 2^−ΔΔ*Ct*^ method, with *GAPDH* as an internal reference. All samples were evaluated three times. The corresponding primers used are listed in Supplementary Table S1.

### 2.9. Statistical Analyses

All statistical analyses were implemented using R (3.6.1 version). The Wilcoxon rank test was used to verify the statistical significance between continuous variables, and the chi-squared test was used to compare classified variables. A *P* value < 0.05 was considered to indicate statistically significant results.

## 3. Results

### 3.1. Immune-Related Genes in Esophageal Carcinoma

In total, 81 tumor and 11 healthy tissue samples from TCGA ESCC were used for differential expression analysis and 4838 differentially expressed genes were screened (false discovery rate < 0.05, ∣log_2_ fold change | >0.585 as a standard). Cross-analysis with immune-related gene databases (Immport, IRIS, and InnateDB) revelated 1271 differentially expressed immune-related genes in the TCGA ESCC dataset. Overall, 713 and 558 genes were up- and downregulated, respectively. GO enrichment and Kyoto Encyclopedia of Genes and Genomes (KEGG) analyses further showed that these differentially expressed immune-related genes were significantly correlated with 479 GO terms and 56 KEGG pathways (Supplementary Figure S1 and Table S2).

Further assessment of the differentially expressed immune-related ESCC genes by WGCNA according to the coexpression patterns revealed eight modules. Based on the correlation coefficient between each gene module and ESCC, genes in the brown and turquoise modules (correlation coefficient with ESCC > 0.7) were selected for further analysis (Supplementary Figure S2).

**Figure 2:**
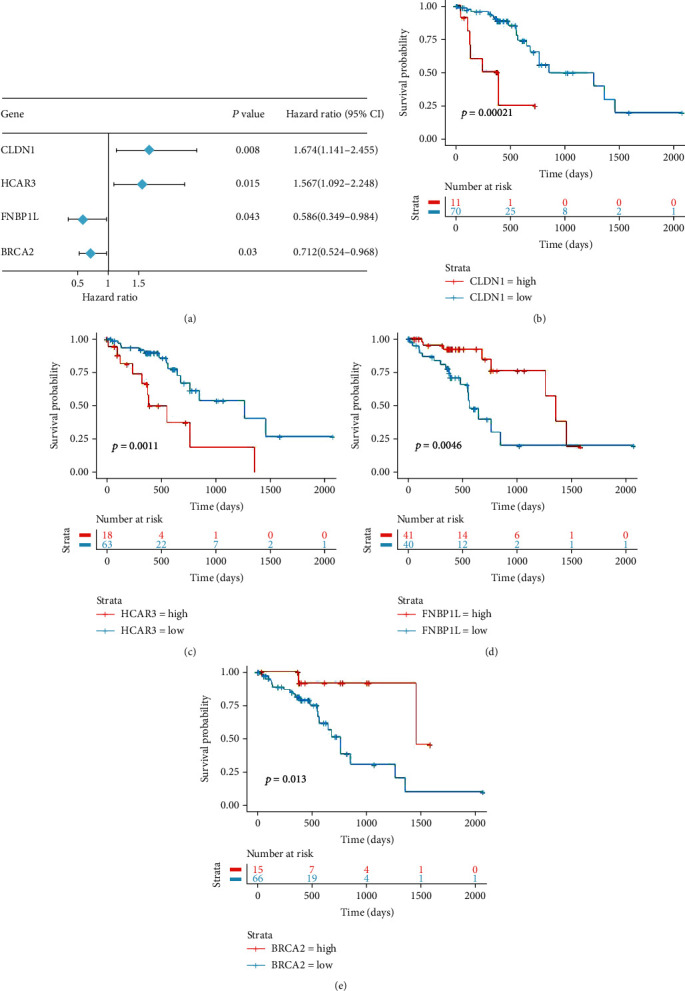
Survival analysis of genes included in the IRGPI. (a) Univariate Cox analysis of immune-related genes in IRGPI with 95% confidence intervals (CI) and hazard ratio (HR). (b–e) Kaplan–Meier survival analysis of each gene comprised in the IRGPI.

The 547 genes in the brown and turquoise modules were analyzed by univariate Cox regression, which revealed that 25 genes were associated with the prognosis of ESCC. By lasso Cox regression, four genes (*CLDN1*, *HCAR3*, *FNBP1L*, and *BRCA2*) were selected as the best model (Supplementary Figure S3). Using the maximally selected test statistics from R package survminer, we determined the best cutoff points for survival analysis of the above four genes. The cutoff points of *CLDN1*, *HCAR3*, *FNBP1L*, and *BRCA2* for survival analysis were 9.39, 2.18, 5.17, and 4.98, respectively. The results showed that high expression of *CLDN1* and *HCAR3* was associated with poor prognosis, whereas high expression of *FNBP1L* and *BRCA2* was associated with good prognosis in the TCGA ESCC cohort (Figure 2).

### 3.2. Prognostic Value of IRGPI in TCGA and the Validation ESCC Cohort

**Figure 3:**
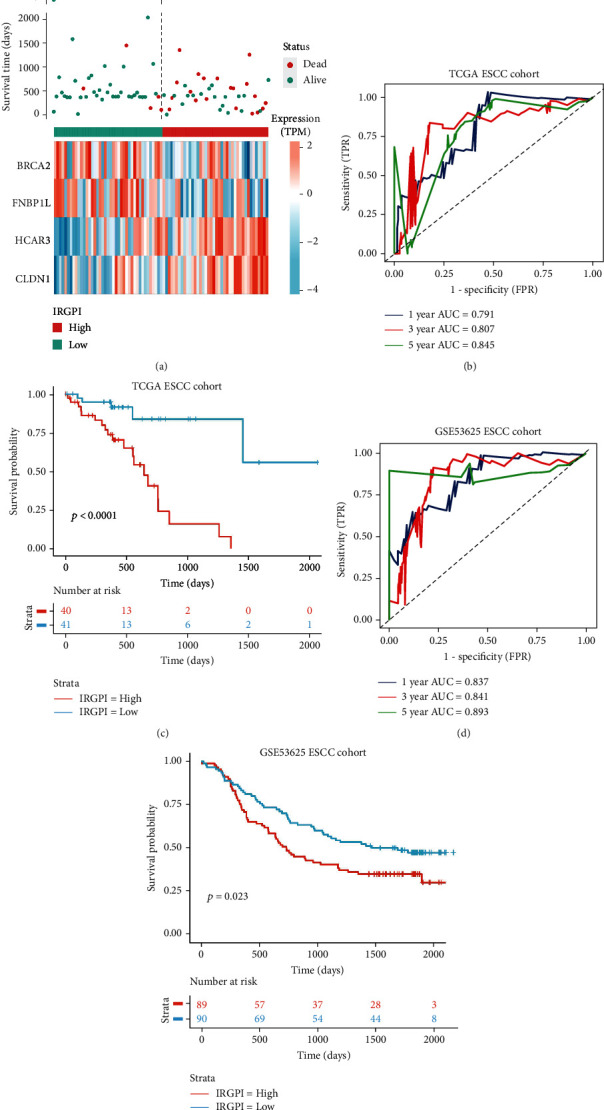
Prognostic value of IRGPI in TCGA ESCC and GSE53625 cohorts. (a) IRGPI score distribution, survival status, and expression of immune-related genes of samples in different IRGPI groups. The *x*-axis represents the individual samples in the ESCC cohort. (b, c) ROC curve and Kaplan–Meier survival analyses of IRGPI in TCGA ESCC cohort. (d, e) ROC curve and Kaplan–Meier survival analyses of IRGPI in the GSE53625 cohort.

We used the aforementioned four genes to construct an immune-related gene prognosis model of ESCC by multivariate Cox regression. The formula for IRGPI was as follows:

(1) $$\begin{array}{c} \;0.40384367\times {\text{Exp}}_{\text{CLDN}1}+0.41720423\times {\text{Exp}}_{\text{HCAR}3}-0.38953082\times {\text{Exp}}_{\text{FNBP}1\text{L}}-0.37979347\times {\text{Exp}}_{\text{BRCA}2}, \end{array}$$

in which the correlation coefficient from the Cox proportional hazard model of each gene was multiplied by the expression of the corresponding gene. The TCGA ESCC cohort was divided into high- and low-IRGPI groups according to the risk score, with the median IRGPI defined as the cutoff point (Figure 3(a)). Using the median IRGPI of 0.13 as the cutoff value, the high- and low-IRGPI groups showed different prognosis outcomes, immune infiltration, mutation characteristics, and responses to chemotherapy and immunotherapy. The expression of *CLDN1* in the high-IRGPI group was higher than that in the low-IRGPI group (Student's *t*-test, *P* < 0.0001), and the median expression levels were 8.56 and 7.4, respectively. The expression of *HCAR3* in the high-IRGPI group was higher than that in the low-IRGPI group (Student's *t*-test, *P* < 0.0001), and the median expression levels were 1.89 and 0.51, respectively. The expression of *FNBP1L* in the high-IRGPI group was lower than that in the low-IRGPI group (Student's *t*-test, *P* < 0.0001), and the median expression levels were 4.90 and 5.48, respectively. The expression of *BRCA2* in the high-IRGPI group was lower than that in the low-IRGPI group (Student's *t*-test, *P* < 0.01), and the median expression levels were 4.13 and 4.65, respectively.

The prognostic value of IRGPI in the TCGA ESCC cohort was evaluated using a time-dependent receiver operating characteristic (ROC) curve. The area under the curve (AUC) values of IRGPI at 1, 3, and 5 years were 0.791, 0.807, and 0.845, respectively (Figure 3(b)). Moreover, Kaplan–Meier analysis showed that the prognosis of the high-IRGPI group in the TCGA ESCC cohort was significantly worse than that of the low-IRGPI group (logrank test, *P* < 0.0001) (Figure 3(c)). The prognostic value of IRGPI was confirmed in the ESCC cohort (GSE53625), for which the AUC values of 1, 3, and 5 years in the ROC curve were 0.837, 0.841, and 0.893, respectively, and the prognosis of the high-IRGPI group was worse than that of the low-IRGPI group (logrank test, *P* < 0.05) (Figures 3(d) and 3(e)).

### 3.3. Different Molecular Characteristics of the IRGPI Subgroups

**Figure 4:**
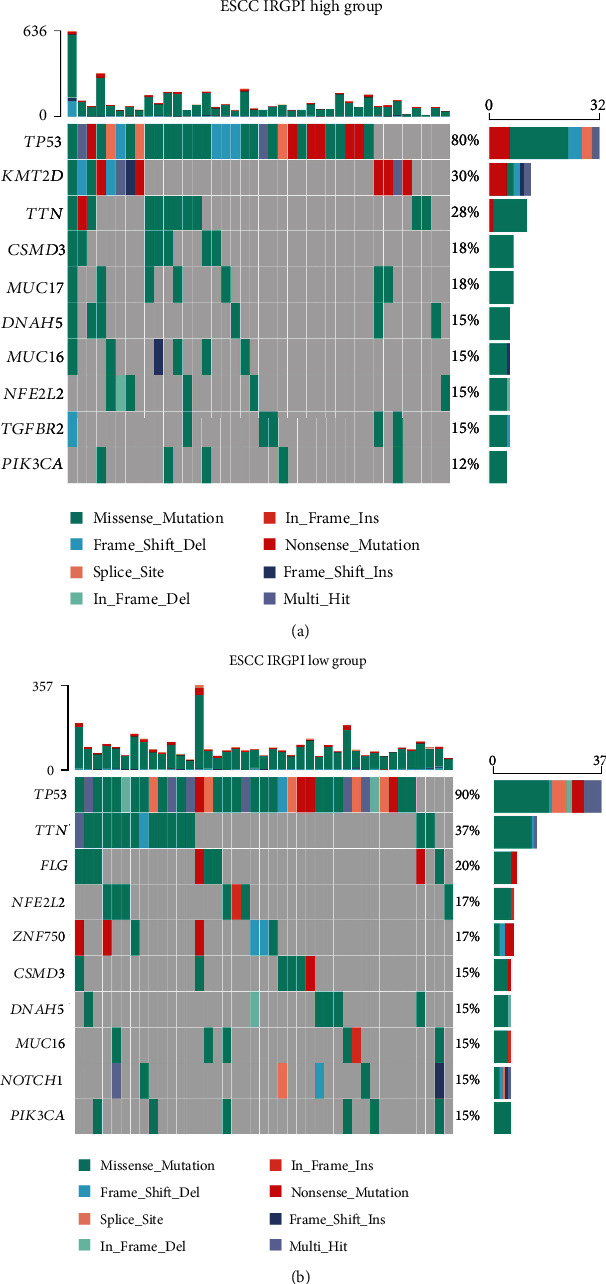
Differences in the mutational status within the IRGPI subgroups. Top 10 mutated genes in the (a) high- and (b) low-IRGPI groups. Points on the *x*-axis represent the samples in each group. Mutated genes are ranked according to the proportion of mutations. The top portion shows the total number of mutations in the ESCC samples, whereas the right shows the proportion of samples with mutations in these genes, and the color is used to indicate the type of mutation.

First, we analyzed the differences in the mutational status of the different IRGPI subgroups. In ESCC, the most common mutation type was a missense mutation, followed by nonsense mutation, and C > T was the most common single-nucleotide variant mutation. In both IRGPI groups, *TP53*, *TTN*, *CSMD3*, *DNAH5*, *MUC16*, *NFE2L2*, and *PIK3CA* were the most commonly mutated genes, showing mutation rates of over 10% in ESCC samples. *KMT2D*, *MUC17*, and *TGFBR2* mutations were more common in the high-IRGPI group, whereas *FLG*, *ZNF750*, and *NOTCH1* mutations were more common in the low-IRGPI group (Figure 4).

**Figure 5:**
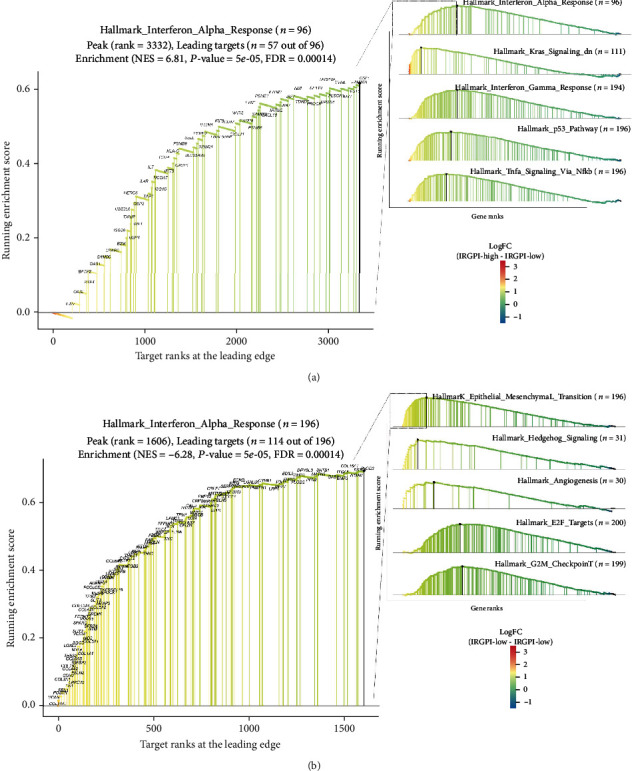
Different gene sets enriched in the IRGPI subgroups. Top five most enriched gene sets in the (a) high- and (b) low-IRGPI groups. The left part of each graph shows the detailed information and leading edge of the most significantly enriched gene set.

Next, GSEA was performed on different IRGPI subgroups. The top five gene sets enriched in the high-IRGPI group were an interferon-*α* response, KRAS signaling DN, interferon-gamma response, P53 pathway, and TNF*α* signaling via NF-*κ*B (Figure 5(a) and Supplementary Table S3). The top five gene sets enriched in the low-IRGPI group were epithelial-mesenchymal transition, hedgehog signaling, angiogenesis, E2F targets, and G2M checkpoint (Figure 5(b)).

### 3.4. Different Immune Characteristics of IRGPI Subgroups

**Figure 6:**
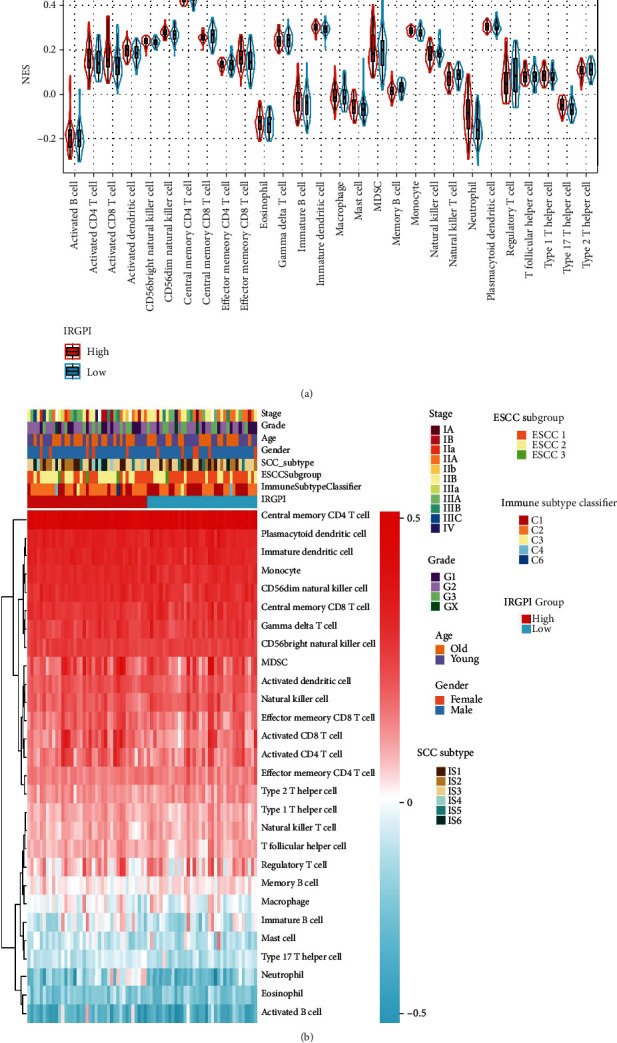
Immune cell infiltration of ESCC samples in the IRGPI subgroups. (a) Wilcoxon rank test was used to inspect the statistical differences of normalized enrichment score (NES) in 28 immune cells between IRGPI subgroups (ns: *P* > 0.05, ^∗^*P* ≤ 0.05, ^∗∗^*P* ≤ 0.01, ^∗∗∗^*P* ≤ 0.001, and ^∗∗∗∗^*P* ≤ 0.0001). The plot shows median (thick lines), quartiles (bottom and top of the boxes), and kernel density estimation (outlines) for each NES distribution. (b) Landscape of the tumor microenvironment in TCGA ESCC subgroups. Clinical features and group information are used as column annotations.

We used the immune cell gene set to analyze immune cell infiltration within the tumor in the TCGA ESCC cohort using the ssGSEA method. The Wilcox rank test was used to distinguish differences in immune cell infiltration between the IRGPI groups (Figure 6(a)).

In the high-IRGPI group, activated CD8 T cells, monocytes, neutrophils, and type 17 T helper cells were highly infiltrated. In the low-IRGPI group, memory B cell infiltration was higher than that in the high-IRGPI group. Additionally, we used the cellular landscape to assess the infiltration of immune cells according to the different clinical features and groups of the samples (Figure 6(b)), including the tumor stage and grade, age, gender, and other tumor classification models.

**Figure 7:**
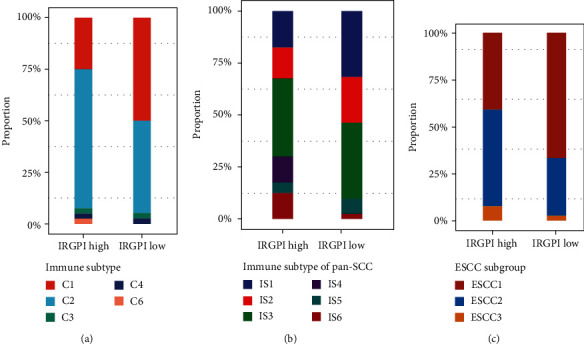
Molecular and immune subtype distribution of IRGPI subgroups. (a) Distribution of immune subtypes (C1-6) between IRGPI subgroups. (b) Distribution of pan-SCC immune subtypes (IS1-6) between IRGPI subgroups. (c) Distribution of ESCC subgroups (ESCC1, ESCC2, and ESCC3) between IRGPI subgroups.

According to the information on the microenvironment and mutation data in TCGA database, tumors were divided into six immune subtypes: C1 (wound healing), C2 (interferon-*γ*-dominant), C3 (inflammatory), C4 (lymphocyte-depleted), C5 (immunologically quiet), and C6 (TGF-*β*-dominant) [35]. Compared with the IRGPI subgroups of the TCGA ESCC cohort, there were more C2 types in the high-IRGPI group and more C1 types in the low-IRGPI group (*P* = 0.04457, chi-squared test) (Figure 7(a)). According to the characteristics of the immune cell microenvironment of TCGA squamous cell carcinoma, these cancers could be divided into six immune subtypes, identified as IS 1–6 [36]. IS4 and IS6 subtypes were more prevalent in the high-IRGPI group than in the low-IRGPI group, and there were more IS1 in the low-IRGPI group (*P* = 0.01081, chi-squared test) (Figure 7(b)). According to the molecular characteristics of ESCC, these samples were divided into three subtypes: ESCC1, ESCC2, and ESCC3 [2]. Overall, the high-IRGPI group contained more ESCC2 samples (*P* = 0.06487, chi-squared test) (Figure 7(c)).

### 3.5. Treatment Strategies Vary between IRGPI Subgroups

**Figure 8:**
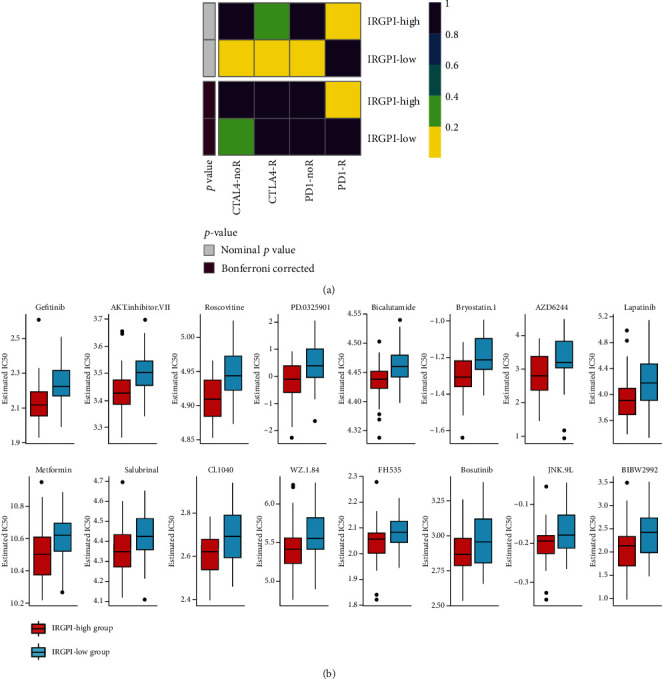
Different responses of IRGPI subgroups to immunotherapy and chemotherapy. (a) Compared with published transcriptome data of melanoma immunotherapy using SubMap, the response of IRGPI subgroups to immunotherapy was predicted. The TCGA ESCC high-IRGPI group may be more sensitive to the PD-1 inhibitor. (b) Based on the transcriptome data of GDSC cell lines and IRGPI subgroups, a ridge regression model was constructed to predict the different estimated IC_50_ values of the drugs. Drugs with a significant difference (*P* < 0.01) between the IRGPI subgroups are shown.

We used SubMap analysis in GenePattern to predict the possible IRGPI subgroup response to immunotherapy by comparing with immunotherapy data of melanoma samples. Overall, the results suggested that anti-PD-1 therapy was more likely to be effective in patients in the high-IRGPI group (*P* = 0.0169830, Bonferroni corrected *P* = 0.1358641) (Figure 8(a)).

Based on the expression profile of GDSC and TCGA ESCC cohorts, we constructed a ridge regression model to predict the IC_50_ of drugs from IRGPI subgroups. Drugs such as gefitinib showed a significant difference in IC_50_ between the IRGPI groups (*P* < 0.001) (Figure 8(b)).

### 3.6. Verification of the Expression of Immune-Related Genes in ESCC

**Figure 9:**
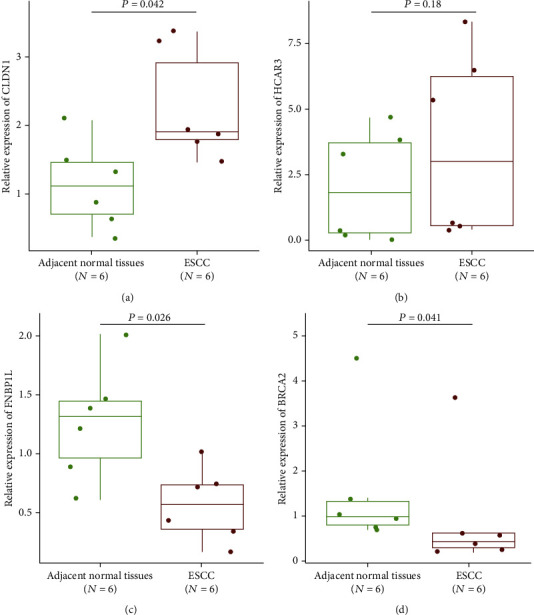
*CLDN1*, *HCAR3*, *FNBP1L*, and *BRCA2* are differently expressed in ESCC. Primary tissue tumor and paracancerous biopsies of six patients with ESCC were evaluated by real-time qPCR. Wilcox test was used to analyze differences between groups.

We verified the expression of genes in the IRGPI in the collected ESCC and adjacent paracancerous tissues. The trend in the qPCR results was the same as that in TCGA ESCC cohorts, confirming the role of these genes in ESCC (Figure 9). The median relative expression of CLDN1 was 1.11 in paracancerous tissues and 1.91 in ESCC tissues, which was significantly increased in tumor tissues (Wilcox test, *P* = 0.042). The median relative expression of HCAR3 was 1.87 in paracancerous tissues and 3.04 in ESCC tissues, which was increased in tumor tissues (Wilcox test, *P* = 0.18). The median relative expression of FNBP1L was 1.31 in paracancerous tissues and 0.57 in ESCC tissues, which was significantly decreased in tumor tissues (Wilcox test, *P* = 0.026). The median relative expression of BRCA2 was 1.04 in paracancerous tissues and 0.46 in ESCC tissues, which was significantly decreased in tumor tissues (Wilcox test, *P* = 0.041).

## 4. Discussion

The therapeutic effect of ICI therapy results from the interactions of the tumor cells, tumor microenvironment, and immune system. Previous clinical trials showed that ICI therapy can effectively prolong the survival time and reduce treatment-related adverse reactions in patients with advanced ESCC compared with traditional chemotherapy and radiotherapy [5, 6, 37]; however, there are uncertainties, such as drug resistance and pseudoprogression, in some populations [11]. Therefore, it is vital to establish a new and robust method for evaluating and predicting the clinical efficacy of ICI therapy in patients with ESCC.

Expression of immune-related genes in tumor samples impacts the tumor immune microenvironment. Thus, an immune gene signature can effectively help predict the clinical benefit of patients receiving immunotherapy [14]. In this study, differentially expressed immune ESCC genes were identified from an immune gene set. The gene modules closely related to the occurrence of ESCC were screened by WGCNA, and genes related to the prognosis of patients with ESCC were further screened with univariate Cox regression and lasso regression. Taken together, we constructed a prognostic index of immune-related genes in ESCC. The IRGPI was demonstrated to be a stable and robust prognostic indicator using publicly available ESCC data, with a high-IRGPI score associated with a poor prognosis. The predictive potential of the IRGPI was confirmed in a second, validation publicly available ESCC dataset.

The IRGPI is based on the expression of four genes: *CLDN1*, *HCAR3*, *FNBP1L*, and *BRCA2*. CLDN1 is a membrane protein involved in the formation of tight junctions between cells and regulates the proliferation and metastasis of various tumors [38], including ESCC by inducing autophagy through the AMPK/STAT1/ULK1 signaling pathway [39], and is closely related to lymphocyte reactions in colorectal cancer [40]. HCAR3 is a member of the G protein-coupled receptor superfamily. Previous studies showed that *HCAR3* is a potential target for regulating cellular immunity and metabolism [41], with its activation by uracil acid exerting an immunosuppressive effect [42, 43]. Moreover, HCAR3 is essential for the metabolism and proliferation of breast cancer cells [44]. FNBP1L is involved in connecting the cell surface signal to the actin cytoskeleton by interacting with CDC42 and N-WASP. FNBP1L promotes epidermal growth factor-induced cell migration and invasion in epidermal and breast cancer [45, 46]. *BRCA2* is a common tumor suppressor gene, and its mutation increases the risk of ESCC [47–49]. In lung and breast cancer cells, long-term induced inactivation of *BRCA2* leads to upregulation of interferon-stimulated genes and activation of the cGAS/STING/STAT pathway, confirming that inactivation of *BRCA2* triggers cellular innate immune responses [50]. In the calculation of IRGPI, the coefficients of *CLDN1* and *HCAR3* were positive, whereas those of *FNBP1L* and *BRCA2* were negative. Therefore, IRGPI is positively correlated with the expression of *CLDN1* and *HCAR3* and negatively correlated with that of *FNBP1L* and *BRCA2*.

To explore the differences in the molecular characteristics between IRGPI subgroups, we analyzed their mutational status. C > T transitions are the most common single-nucleotide variant type of ESCC. A high frequency of C > T substitution may be associated with CpG methylation, and the change of germ line methylation can lead to substitution rate variation at the CpG region [51]. The most common mutation type in the two subgroups was missense mutations, followed by nonsense mutations. *KMT2D* mutation was more common in the high-IRGPI group. It has been reported that *KMT2D* mutation is the main modulator of ICI in several tumors [52], as *KMT2D* mutation enhances the immune infiltration and immunogenicity of tumors, thereby making tumors more sensitive to ICI therapy. In turn, *ZNF750* mutation was more common in the low-IRGPI group. Studies have shown that *ZNF750* is a commonly mutated gene in ESCC, mainly with nonsense mutations. *ZNF750* can inhibit epithelial-mesenchymal transition by directly depressing the *SNAI1* promoter [53]. In addition, a decrease in ZNF750 levels promotes angiogenesis in ESCC by activating the DANCR/miR-4707-3p/FOXC2 axis [54]. Therefore, analysis of the mutation information of the IRGPI subgroups suggested that patients with high-IRGPI scores are more sensitive to ICI treatment and that those with low-IRGPI values are more prone to the epithelial-mesenchymal transition and angiogenesis phenotype.

GSEA revealed that the enriched gene sets differed between the IRGPI subgroups. The interferon-*α* response gene set was found to be enriched in the high-IRGPI group. Interferon-*α* can effectively activate the immune response and reverse the immunosuppressive effect of mesenchymal stem cells [55, 56]. Additionally, the interferon-*γ* response gene set was enriched in the high-IRGPI group. Interferon-*γ* maintains immune homeostasis in the tumor microenvironment, limits adaptive and innate immune killing, and, thus, limits the response of patients to ICI treatment [57, 58]. The high-IRGPI group was enriched in the KRAS signaling pathway gene set, which can affect the immune escape of tumor cells [59]. Furthermore, the P53 and NF-*κ*B pathways, which play a role in the tumor immune microenvironment and ICI therapy, were enriched in the high-IRGPI group [60, 61]. The epithelial-mesenchymal transition, angiogenesis, and E2F gene sets were enriched in the low-IRGPI group, further suggesting that this group is prone to tumor invasion, metastasis, and cell cycle. Therefore, the gene enrichment results suggest that high-IRGPI values are associated with tumor immunity, in contrast to the low-IRGPI group.

Next, we analyzed the difference in immune cell infiltration between the IRGPI subgroups. In the high-IRGPI group, activated CD8 T cells, monocytes, neutrophils, and type 17 T helper cells showed higher infiltration, whereas in the low-IRGPI group, memory B cell infiltration was more common. Studies have shown that CD8 T cells are closely related to the expression of PD-L1, suggesting their value for predicting the prognosis of patients and response to ICI treatment [62–64]. The increase in the neutrophil count in tumors is often closely related to poor prognosis, explaining the poor prognosis of patients in the high-IRGPI group [65]. Interleukin-17, produced by type 17 T helper cells, stimulates tumor and stroma cells to produce tumor-promoting factors, whereas interleukin-8, produced by type 17 T helper cells, recruits neutrophils [66]. Memory B cell infiltration was higher in the low-IRGPI group. A previous study showed that the new subgroup of memory B cells can promote angiogenesis [67]. Therefore, analysis of immune cell infiltration in the IRGPI subgroups showed that under the effect of immune-infiltrating cells, high-IRGPI values were indicative of adverse prognosis and predicted the ICI response, whereas low-IRGPI indicated angiogenesis.

Combined with other immune subtypes, we can determine the immune status of IRGPI subgroups. Compared with pan-cancer immune subtypes, there were more C2-type patients in the high-IRGPI group and more C1-type patients in the low-IRGPI group [35]. The C2 type is characterized by an IFN-*γ* response and high CD8 T cell markers and lymphocyte infiltration rate, indicating a better immune response but poor prognosis. The C1 type indicates more angiogenic gene expression. In squamous cell carcinoma immune subtypes, IS4 and IS6 were more prevalent in the high-IRGPI group than in the low-IRGPI group [36]. IS4 showed the highest T cell expression and IFN-*γ* response, with a good immune activation phenotype, whereas IS1 showed an immunosuppressive phenotype. The high-IRGPI group tended to undergo ESCC2 classification, which involves higher leukocyte infiltration [2]. Overall, the immunophenotype of the high-IRGPI group was more active than that of the low-IRGPI group.

## 5. Conclusions

Traditional chemotherapy and radiotherapy are effective in few patients with ESCC. Hence, for these patients, ICI therapy may be beneficial for prolonging their survival time and reducing the incidence of treatment-related adverse reactions. Taken together, this study may fill the gap in the need for new biomarkers and the proposed IRGPI may be used as a biomarker to evaluate ESCC prognosis and the response to ICI therapy.

## Data Availability

The datasets generated and analyzed during the current study are available in The Cancer Genome Atlas (TCGA) (https://portal.gdc.cancer.gov) and the Gene Expression Omnibus (GEO) (http://www.ncbi.nlm.nih.gov/geo).

## Supplementary Materials

Supplementary Materials Supplementary Figure S1: Gene ontology (GO) enrichment analysis of differentially expressed immune-related genes in the TCGA ESCC cohort. Supplementary Figure S2: WGCNA method used to identify the hub genes. (A) Determination of soft threshold in WGCNA. (B) WGCNA analysis of differentially expressed immune-related genes in ESCC and the gene modules. (C) Gene expression clustering tree and coexpression topological heat map. (D) Relationship between the gene modules and features of ESCC. Supplementary Figure S3: differentially expressed immune-related genes analyzed using lasso Cox regression to determine genes included in the prognostic model. (A) Lasso Cox regression fitting process. (B) Tenfold crossvalidation was used to determine the *λ* values. The lambda value in lasso Cox reflects the degree of regularization. The larger the lambda value, the fewer effective variables are screened. Tenfold crossvalidation was used to determine the lambda values; the minimum crossvalidation error is set to the best lambda value. Supplementary Table S1: primers used for qPCR. Supplementary Table S2: GO terms and KEGG pathway enrichment analyses of differentially expressed immune-related genes of ESCC. Supplementary Table S3: GSEA enrichment analysis between IRGPI subgroups.
