# Supplementary material for: Construction and Validation of an Immune-Related Gene Prognostic Index for Esophageal Squamous Cell Carcinoma
Source: Biomed Res Int. 2021 Oct 21;2021:7430315. doi: 10.1155/2021/7430315 (PMC8553461; doi:10.1155/2021/7430315)
Supplement: Supplementary Materials — Supplementary Figure S1: Gene ontology (GO) enrichment analysis of differentially expressed immune-related genes in the TCGA ESCC cohort. Supplementary Figure S2: WGCNA method used to identify the hub genes. (A) Determination of soft threshold in WGCNA. (B) WGCNA analysis of differentially expressed immune-related genes in ESCC and the gene modules. (C) Gene expression clustering tree and coexpression topological heat map. (D) Relationship between the gene modules and features of ESCC. Supplementary Figure S3: differentially expressed immune-related genes analyzed using lasso Cox regression to determine genes included in the prognostic model. (A) Lasso Cox regression fitting process. (B) Tenfold crossvalidation was used to determine the λ values. The lambda value in lasso Cox reflects the degree of regularization. The larger the lambda value, the fewer effective variables are screened. Tenfold crossvalidation was used to determine the lambda values; the minimum crossvalidation error is set to the best lambda value. Supplementary Table S1: primers used for qPCR. Supplementary Table S2: GO terms and KEGG pathway enrichment analyses of differentially expressed immune-related genes of ESCC. Supplementary Table S3: GSEA enrichment analysis between IRGPI subgroups. [file 7430315.f1.zip › Table S2 .docx]

| **Category** | **Terms** | **Counts** | **GeneRatio** | **pValue** | **FDR** | **foldEnrichment** |
| --- | --- | --- | --- | --- | --- | --- |
| GO_BP | GO:2000116~regulation of cysteine-type endopeptidase activity | 83 | 83/1250 | 2.97E-37 | 1.77E-33 | 5.0677831 |
| GO_BP | GO:0050900~leukocyte migration | 108 | 108/1250 | 8.33E-33 | 9.90E-30 | 3.5940804 |
| GO_BP | GO:0002237~response to molecule of bacterial origin | 94 | 94/1250 | 1.16E-32 | 1.15E-29 | 4.0074036 |
| GO_BP | GO:0045088~regulation of innate immune response | 108 | 108/1250 | 2.08E-31 | 1.41E-28 | 3.4737429 |
| GO_BP | GO:0045785~positive regulation of cell adhesion | 101 | 101/1250 | 2.72E-31 | 1.62E-28 | 3.6475429 |
| GO_BP | GO:0001819~positive regulation of cytokine production | 108 | 108/1250 | 1.33E-30 | 6.58E-28 | 3.4053322 |
| GO_BP | GO:0042110~T cell activation | 108 | 108/1250 | 3.63E-30 | 1.66E-27 | 3.3684779 |
| GO_BP | GO:0043062~extracellular structure organization | 101 | 101/1250 | 1.68E-29 | 6.69E-27 | 3.4817455 |
| GO_BP | GO:0097193~intrinsic apoptotic signaling pathway | 82 | 82/1250 | 1.69E-29 | 6.69E-27 | 4.1170286 |
| GO_BP | GO:1903706~regulation of hemopoiesis | 108 | 108/1250 | 2.13E-29 | 7.91E-27 | 3.3041121 |
| GO_BP | GO:0048732~gland development | 101 | 101/1250 | 3.59E-28 | 1.19E-25 | 3.3611307 |
| GO_BP | GO:0031589~cell-substrate adhesion | 87 | 87/1250 | 1.20E-26 | 3.56E-24 | 3.6024 |
| GO_BP | GO:0051249~regulation of lymphocyte activation | 95 | 95/1250 | 6.90E-26 | 1.71E-23 | 3.2985831 |
| GO_BP | GO:0051047~positive regulation of secretion | 96 | 96/1250 | 1.60E-25 | 3.81E-23 | 3.239629 |
| GO_BP | GO:0032103~positive regulation of response to external stimulus | 80 | 80/1250 | 4.58E-25 | 1.05E-22 | 3.659581 |
| GO_BP | GO:0002683~negative regulation of immune system process | 97 | 97/1250 | 6.12E-24 | 1.17E-21 | 3.0719367 |
| GO_BP | GO:0071356~cellular response to tumor necrosis factor | 74 | 74/1250 | 8.48E-24 | 1.53E-21 | 3.728358 |
| GO_BP | GO:0050878~regulation of body fluid levels | 102 | 102/1250 | 8.79E-24 | 1.54E-21 | 2.9573022 |
| GO_BP | GO:0051090~regulation of DNA-binding transcription factor activity | 93 | 93/1250 | 9.06E-24 | 1.54E-21 | 3.1457577 |
| GO_BP | GO:0050727~regulation of inflammatory response | 91 | 91/1250 | 9.77E-24 | 1.61E-21 | 3.1904467 |
| GO_BP | GO:0048863~stem cell differentiation | 69 | 69/1250 | 1.77E-23 | 2.69E-21 | 3.8990682 |
| GO_BP | GO:0034612~response to tumor necrosis factor | 76 | 76/1250 | 3.24E-23 | 4.81E-21 | 3.5788549 |
| GO_BP | GO:0033209~tumor necrosis factor-mediated signaling pathway | 54 | 54/1250 | 4.78E-23 | 6.93E-21 | 4.7446244 |
| GO_BP | GO:0050673~epithelial cell proliferation | 90 | 90/1250 | 1.15E-22 | 1.58E-20 | 3.1099856 |
| GO_BP | GO:0030099~myeloid cell differentiation | 88 | 88/1250 | 7.31E-22 | 9.24E-20 | 3.0777786 |
| GO_BP | GO:1904951~positive regulation of establishment of protein localization | 93 | 93/1250 | 7.68E-22 | 9.50E-20 | 2.9648071 |
| GO_BP | GO:0050863~regulation of T cell activation | 74 | 74/1250 | 2.36E-21 | 2.75E-19 | 3.4176615 |
| GO_BP | GO:0007159~leukocyte cell-cell adhesion | 76 | 76/1250 | 6.57E-21 | 7.37E-19 | 3.2985831 |
| GO_BP | GO:0070661~leukocyte proliferation | 71 | 71/1250 | 9.65E-21 | 1.03E-18 | 3.4447192 |
| GO_BP | GO:0071216~cellular response to biotic stimulus | 61 | 61/1250 | 2.61E-20 | 2.68E-18 | 3.8051325 |
| GO_BP | GO:0002685~regulation of leukocyte migration | 55 | 55/1250 | 3.18E-20 | 3.21E-18 | 4.12775 |
| GO_BP | GO:0007596~blood coagulation | 75 | 75/1250 | 3.31E-20 | 3.28E-18 | 3.2454054 |
| GO_BP | GO:0050817~coagulation | 75 | 75/1250 | 6.96E-20 | 6.78E-18 | 3.2068843 |
| GO_BP | GO:0070482~response to oxygen levels | 81 | 81/1250 | 1.73E-19 | 1.56E-17 | 3.0004566 |
| GO_BP | GO:0050920~regulation of chemotaxis | 57 | 57/1250 | 1.83E-19 | 1.62E-17 | 3.8742792 |
| GO_BP | GO:0050678~regulation of epithelial cell proliferation | 77 | 77/1250 | 3.17E-19 | 2.69E-17 | 3.0735158 |
| GO_BP | GO:0043405~regulation of MAP kinase activity | 73 | 73/1250 | 9.27E-19 | 7.35E-17 | 3.1306571 |
| GO_BP | GO:0050663~cytokine secretion | 59 | 59/1250 | 1.25E-18 | 9.48E-17 | 3.6331897 |
| GO_BP | GO:0016049~cell growth | 90 | 90/1250 | 1.28E-18 | 9.51E-17 | 2.7245042 |
| GO_BP | GO:0032102~negative regulation of response to external stimulus | 75 | 75/1250 | 1.67E-18 | 1.16E-16 | 3.0442817 |
| GO_BP | GO:0071559~response to transforming growth factor beta | 61 | 61/1250 | 3.47E-18 | 2.37E-16 | 3.4742514 |
| GO_BP | GO:0071560~cellular response to transforming growth factor beta stimulus | 60 | 60/1250 | 4.47E-18 | 3.02E-16 | 3.5003077 |
| GO_BP | GO:0042119~neutrophil activation | 91 | 91/1250 | 6.27E-18 | 4.16E-16 | 2.6436968 |
| GO_BP | GO:0002460~adaptive immune response based on somatic recombination | 61 | 61/1250 | 6.43E-18 | 4.20E-16 | 3.4335375 |
| GO_BP | GO:0002446~neutrophil mediated immunity | 91 | 91/1250 | 7.17E-18 | 4.63E-16 | 2.6383775 |
| GO_BP | GO:0001818~negative regulation of cytokine production | 65 | 65/1250 | 1.22E-17 | 7.50E-16 | 3.2409135 |
| GO_BP | GO:0030100~regulation of endocytosis | 63 | 63/1250 | 2.63E-17 | 1.49E-15 | 3.2654849 |
| GO_BP | GO:0006909~phagocytosis | 61 | 61/1250 | 3.16E-17 | 1.74E-15 | 3.3294909 |
| GO_BP | GO:0061041~regulation of wound healing | 43 | 43/1250 | 4.37E-17 | 2.38E-15 | 4.3634704 |
| GO_BP | GO:0002283~neutrophil activation involved in immune response | 88 | 88/1250 | 5.28E-17 | 2.80E-15 | 2.6091457 |
| GO_BP | GO:0043312~neutrophil degranulation | 87 | 87/1250 | 1.12E-16 | 5.71E-15 | 2.595518 |
| GO_BP | GO:0018108~peptidyl-tyrosine phosphorylation | 72 | 72/1250 | 1.58E-16 | 7.95E-15 | 2.8899476 |
| GO_BP | GO:0098742~cell-cell adhesion via plasma-membrane adhesion molecules | 61 | 61/1250 | 2.09E-16 | 1.01E-14 | 3.2079766 |
| GO_BP | GO:0001558~regulation of cell growth | 78 | 78/1250 | 2.23E-16 | 1.07E-14 | 2.7346686 |
| GO_BP | GO:0098542~defense response to other organism | 88 | 88/1250 | 2.49E-16 | 1.18E-14 | 2.5462747 |
| GO_BP | GO:0045926~negative regulation of growth | 57 | 57/1250 | 4.52E-16 | 2.03E-14 | 3.3118839 |
| GO_BP | GO:1905330~regulation of morphogenesis of an epithelium | 47 | 47/1250 | 7.63E-16 | 3.32E-14 | 3.7835263 |
| GO_BP | GO:0060562~epithelial tube morphogenesis | 66 | 66/1250 | 8.24E-16 | 3.52E-14 | 2.9627215 |
| GO_BP | GO:0032602~chemokine production | 32 | 32/1250 | 8.69E-16 | 3.69E-14 | 5.3000828 |
| GO_BP | GO:0042326~negative regulation of phosphorylation | 81 | 81/1250 | 2.03E-15 | 8.32E-14 | 2.576551 |
| GO_BP | GO:2000736~regulation of stem cell differentiation | 36 | 36/1250 | 4.96E-15 | 1.95E-13 | 4.5108313 |
| GO_BP | GO:0048872~homeostasis of number of cells | 55 | 55/1250 | 5.27E-15 | 2.06E-13 | 3.2216585 |
| GO_BP | GO:0051271~negative regulation of cellular component movement | 68 | 68/1250 | 7.91E-15 | 3.03E-13 | 2.7836727 |
| GO_BP | GO:0010634~positive regulation of epithelial cell migration | 43 | 43/1250 | 1.09E-14 | 3.99E-13 | 3.8013055 |
| GO_BP | GO:0007050~cell cycle arrest | 53 | 53/1250 | 1.13E-14 | 4.12E-13 | 3.2498247 |
| GO_BP | GO:0002449~lymphocyte mediated immunity | 54 | 54/1250 | 2.52E-14 | 8.71E-13 | 3.1502769 |
| GO_BP | GO:0071453~cellular response to oxygen levels | 51 | 51/1250 | 5.10E-14 | 1.68E-12 | 3.2232 |
| GO_BP | GO:0001101~response to acid chemical | 65 | 65/1250 | 5.24E-14 | 1.72E-12 | 2.7547765 |
| GO_BP | GO:0070997~neuron death | 66 | 66/1250 | 5.71E-14 | 1.85E-12 | 2.7250246 |
| GO_BP | GO:0060759~regulation of response to cytokine stimulus | 45 | 45/1250 | 9.88E-14 | 3.04E-12 | 3.4675508 |
| GO_BP | GO:0006979~response to oxidative stress | 75 | 75/1250 | 2.31E-13 | 6.67E-12 | 2.4673973 |
| GO_BP | GO:0072593~reactive oxygen species metabolic process | 56 | 56/1250 | 2.74E-13 | 7.67E-12 | 2.9131321 |
| GO_BP | GO:0061448~connective tissue development | 55 | 55/1250 | 3.23E-13 | 8.97E-12 | 2.9352889 |
| GO_BP | GO:0019882~antigen processing and presentation | 49 | 49/1250 | 3.28E-13 | 8.97E-12 | 3.166235 |
| GO_BP | GO:0033002~muscle cell proliferation | 49 | 49/1250 | 3.28E-13 | 8.97E-12 | 3.166235 |
| GO_BP | GO:0006898~receptor-mediated endocytosis | 53 | 53/1250 | 4.45E-13 | 1.20E-11 | 2.9832375 |
| GO_BP | GO:0034341~response to interferon-gamma | 45 | 45/1250 | 4.89E-13 | 1.32E-11 | 3.3252923 |
| GO_BP | GO:0045787~positive regulation of cell cycle | 68 | 68/1250 | 7.04E-13 | 1.83E-11 | 2.5450722 |
| GO_BP | GO:0040013~negative regulation of locomotion | 65 | 65/1250 | 1.37E-12 | 3.45E-11 | 2.5731429 |
| GO_BP | GO:0034599~cellular response to oxidative stress | 56 | 56/1250 | 1.70E-12 | 4.19E-11 | 2.7921716 |
| GO_BP | GO:1901990~regulation of mitotic cell cycle phase transition | 72 | 72/1250 | 2.42E-12 | 5.83E-11 | 2.4071722 |
| GO_BP | GO:0007409~axonogenesis | 76 | 76/1250 | 2.53E-12 | 6.05E-11 | 2.3400205 |
| GO_BP | GO:0007568~aging | 58 | 58/1250 | 4.71E-12 | 1.07E-10 | 2.6701495 |
| GO_BP | GO:1901214~regulation of neuron death | 58 | 58/1250 | 6.18E-12 | 1.39E-10 | 2.6531962 |
| GO_BP | GO:2000377~regulation of reactive oxygen species metabolic process | 42 | 42/1250 | 9.09E-12 | 1.96E-10 | 3.219166 |
| GO_BP | GO:0048871~multicellular organismal homeostasis | 76 | 76/1250 | 1.01E-11 | 2.17E-10 | 2.2767767 |
| GO_BP | GO:0051348~negative regulation of transferase activity | 53 | 53/1250 | 1.12E-11 | 2.37E-10 | 2.7570715 |
| GO_BP | GO:0046677~response to antibiotic | 58 | 58/1250 | 1.20E-11 | 2.55E-10 | 2.61174 |
| GO_BP | GO:0000302~response to reactive oxygen species | 46 | 46/1250 | 1.79E-11 | 3.70E-10 | 2.9723839 |
| GO_BP | GO:0043900~regulation of multi-organism process | 67 | 67/1250 | 1.99E-11 | 4.07E-10 | 2.3897109 |
| GO_BP | GO:0048660~regulation of smooth muscle cell proliferation | 37 | 37/1250 | 2.00E-11 | 4.07E-10 | 3.439711 |
| GO_BP | GO:0035690~cellular response to drug | 62 | 62/1250 | 2.24E-11 | 4.51E-10 | 2.4816533 |
| GO_BP | GO:0050769~positive regulation of neurogenesis | 74 | 74/1250 | 2.26E-11 | 4.52E-10 | 2.2687455 |
| GO_BP | GO:1905477~positive regulation of protein localization to membrane | 32 | 32/1250 | 2.56E-11 | 5.03E-10 | 3.8108033 |
| GO_BP | GO:0097305~response to alcohol | 47 | 47/1250 | 2.88E-11 | 5.62E-10 | 2.8942359 |
| GO_BP | GO:0019058~viral life cycle | 58 | 58/1250 | 2.98E-11 | 5.80E-10 | 2.5558312 |
| GO_BP | GO:0022604~regulation of cell morphogenesis | 75 | 75/1250 | 3.97E-11 | 7.59E-10 | 2.2282887 |
| GO_BP | GO:0030193~regulation of blood coagulation | 25 | 25/1250 | 4.09E-11 | 7.75E-10 | 4.6184615 |
| GO_BP | GO:0055074~calcium ion homeostasis | 73 | 73/1250 | 5.35E-11 | 1.00E-09 | 2.2428588 |
| GO_BP | GO:1900046~regulation of hemostasis | 25 | 25/1250 | 5.60E-11 | 1.04E-09 | 4.56 |
| GO_BP | GO:0071229~cellular response to acid chemical | 43 | 43/1250 | 6.43E-11 | 1.18E-09 | 2.9932986 |
| GO_BP | GO:0002440~production of molecular mediator of immune response | 42 | 42/1250 | 7.46E-11 | 1.35E-09 | 3.026016 |
| GO_BP | GO:0051701~interaction with host | 43 | 43/1250 | 8.91E-11 | 1.61E-09 | 2.9646545 |
| GO_BP | GO:0006874~cellular calcium ion homeostasis | 71 | 71/1250 | 9.74E-11 | 1.75E-09 | 2.2436 |
| GO_BP | GO:0050918~positive chemotaxis | 23 | 23/1250 | 1.00E-10 | 1.79E-09 | 4.8032 |
| GO_BP | GO:0003158~endothelium development | 32 | 32/1250 | 1.01E-10 | 1.80E-09 | 3.6307654 |
| GO_BP | GO:0050818~regulation of coagulation | 25 | 25/1250 | 1.03E-10 | 1.82E-09 | 4.4474074 |
| GO_BP | GO:0046718~viral entry into host cell | 31 | 31/1250 | 1.26E-10 | 2.19E-09 | 3.6917157 |
| GO_BP | GO:1903829~positive regulation of cellular protein localization | 56 | 56/1250 | 1.43E-10 | 2.47E-09 | 2.5060174 |
| GO_BP | GO:0042063~gliogenesis | 52 | 52/1250 | 1.62E-10 | 2.78E-09 | 2.6017333 |
| GO_BP | GO:0009612~response to mechanical stimulus | 42 | 42/1250 | 3.72E-10 | 6.08E-09 | 2.88192 |
| GO_BP | GO:0008637~apoptotic mitochondrial changes | 30 | 30/1250 | 3.82E-10 | 6.19E-09 | 3.6326723 |
| GO_BP | GO:0090066~regulation of anatomical structure size | 74 | 74/1250 | 4.22E-10 | 6.76E-09 | 2.1326208 |
| GO_BP | GO:0010565~regulation of cellular ketone metabolic process | 38 | 38/1250 | 5.07E-10 | 7.96E-09 | 3.0420267 |
| GO_BP | GO:0110110~positive regulation of animal organ morphogenesis | 24 | 24/1250 | 6.22E-10 | 9.53E-09 | 4.2695111 |
| GO_BP | GO:0002526~acute inflammatory response | 35 | 35/1250 | 6.22E-10 | 9.53E-09 | 3.192 |
| GO_BP | GO:0051098~regulation of binding | 60 | 60/1250 | 7.79E-10 | 1.18E-08 | 2.3178981 |
| GO_BP | GO:0048144~fibroblast proliferation | 24 | 24/1250 | 8.22E-10 | 1.24E-08 | 4.2174439 |
| GO_BP | GO:0060560~developmental growth involved in morphogenesis | 44 | 44/1250 | 9.69E-10 | 1.44E-08 | 2.7211262 |
| GO_BP | GO:0042176~regulation of protein catabolic process | 60 | 60/1250 | 1.19E-09 | 1.74E-08 | 2.293305 |
| GO_BP | GO:0046879~hormone secretion | 53 | 53/1250 | 1.35E-09 | 1.94E-08 | 2.4321936 |
| GO_BP | GO:0000086~G2/M transition of mitotic cell cycle | 45 | 45/1250 | 1.38E-09 | 1.97E-08 | 2.6575082 |
| GO_BP | GO:0001909~leukocyte mediated cytotoxicity | 27 | 27/1250 | 1.79E-09 | 2.52E-08 | 3.7053257 |
| GO_BP | GO:0048640~negative regulation of developmental growth | 28 | 28/1250 | 1.80E-09 | 2.53E-08 | 3.6024 |
| GO_BP | GO:0006521~regulation of cellular amino acid metabolic process | 20 | 20/1250 | 2.31E-09 | 3.19E-08 | 4.724459 |
| GO_BP | GO:0034446~substrate adhesion-dependent cell spreading | 26 | 26/1250 | 2.76E-09 | 3.77E-08 | 3.746496 |
| GO_BP | GO:0001906~cell killing | 35 | 35/1250 | 2.98E-09 | 4.04E-08 | 3.019976 |
| GO_BP | GO:0031145~anaphase-promoting complex-dependent catabolic process | 23 | 23/1250 | 3.54E-09 | 4.72E-08 | 4.0916148 |
| GO_BP | GO:0009914~hormone transport | 53 | 53/1250 | 4.20E-09 | 5.54E-08 | 2.3571259 |
| GO_BP | GO:0062012~regulation of small molecule metabolic process | 67 | 67/1250 | 4.26E-09 | 5.60E-08 | 2.1125672 |
| GO_BP | GO:0030278~regulation of ossification | 38 | 38/1250 | 4.68E-09 | 6.11E-08 | 2.822499 |
| GO_BP | GO:1905475~regulation of protein localization to membrane | 37 | 37/1250 | 4.80E-09 | 6.25E-08 | 2.8664258 |
| GO_BP | GO:0010165~response to X-ray | 14 | 14/1250 | 4.84E-09 | 6.27E-08 | 6.5075613 |
| GO_BP | GO:0010822~positive regulation of mitochondrion organization | 28 | 28/1250 | 5.17E-09 | 6.65E-08 | 3.4484513 |
| GO_BP | GO:0048545~response to steroid hormone | 59 | 59/1250 | 6.99E-09 | 8.68E-08 | 2.2082244 |
| GO_BP | GO:0031348~negative regulation of defense response | 42 | 42/1250 | 7.51E-09 | 9.27E-08 | 2.6199273 |
| GO_BP | GO:0061013~regulation of mRNA catabolic process | 38 | 38/1250 | 9.72E-09 | 1.16E-07 | 2.7515819 |
| GO_BP | GO:0043434~response to peptide hormone | 64 | 64/1250 | 9.90E-09 | 1.17E-07 | 2.1103304 |
| GO_BP | GO:0043112~receptor metabolic process | 37 | 37/1250 | 1.02E-08 | 1.20E-07 | 2.7913885 |
| GO_BP | GO:0048588~developmental cell growth | 41 | 41/1250 | 1.38E-08 | 1.59E-07 | 2.602615 |
| GO_BP | GO:0097345~mitochondrial outer membrane permeabilization | 18 | 18/1250 | 1.51E-08 | 1.72E-07 | 4.7158691 |
| GO_BP | GO:0002562~somatic diversification of immune receptors via germline recombination | 19 | 19/1250 | 2.04E-08 | 2.26E-07 | 4.4158452 |
| GO_BP | GO:0016444~somatic cell DNA recombination | 19 | 19/1250 | 2.04E-08 | 2.26E-07 | 4.4158452 |
| GO_BP | GO:0090257~regulation of muscle system process | 43 | 43/1250 | 2.15E-08 | 2.37E-07 | 2.4984387 |
| GO_BP | GO:2001057~reactive nitrogen species metabolic process | 22 | 22/1250 | 2.43E-08 | 2.67E-07 | 3.8659902 |
| GO_BP | GO:0032970~regulation of actin filament-based process | 57 | 57/1250 | 2.73E-08 | 2.95E-07 | 2.16144 |
| GO_BP | GO:1905710~positive regulation of membrane permeability | 19 | 19/1250 | 2.74E-08 | 2.95E-07 | 4.3457524 |
| GO_BP | GO:0007548~sex differentiation | 45 | 45/1250 | 3.49E-08 | 3.68E-07 | 2.4016 |
| GO_BP | GO:0043487~regulation of RNA stability | 35 | 35/1250 | 3.55E-08 | 3.74E-07 | 2.7559344 |
| GO_BP | GO:0099504~synaptic vesicle cycle | 36 | 36/1250 | 3.87E-08 | 4.01E-07 | 2.7018 |
| GO_BP | GO:0002718~regulation of cytokine production involved in immune response | 22 | 22/1250 | 3.92E-08 | 4.05E-07 | 3.7739429 |
| GO_BP | GO:0006809~nitric oxide biosynthetic process | 20 | 20/1250 | 9.16E-08 | 8.96E-07 | 3.8944865 |
| GO_BP | GO:0010718~positive regulation of epithelial to mesenchymal transition | 16 | 16/1250 | 9.87E-08 | 9.56E-07 | 4.7051755 |
| GO_BP | GO:0090559~regulation of membrane permeability | 21 | 21/1250 | 1.21E-07 | 1.15E-06 | 3.6902634 |
| GO_BP | GO:0060263~regulation of respiratory burst | 9 | 9/1250 | 1.24E-07 | 1.17E-06 | 8.64576 |
| GO_BP | GO:0050803~regulation of synapse structure or activity | 39 | 39/1250 | 1.24E-07 | 1.17E-06 | 2.4756582 |
| GO_BP | GO:0097237~cellular response to toxic substance | 40 | 40/1250 | 1.41E-07 | 1.31E-06 | 2.432 |
| GO_BP | GO:0043500~muscle adaptation | 25 | 25/1250 | 1.46E-07 | 1.36E-06 | 3.2164286 |
| GO_BP | GO:0031334~positive regulation of protein complex assembly | 43 | 43/1250 | 1.53E-07 | 1.41E-06 | 2.3381615 |
| GO_BP | GO:0051235~maintenance of location | 49 | 49/1250 | 2.19E-07 | 1.98E-06 | 2.1725243 |
| GO_BP | GO:0003300~cardiac muscle hypertrophy | 22 | 22/1250 | 2.77E-07 | 2.47E-06 | 3.4087226 |
| GO_BP | GO:1904062~regulation of cation transmembrane transport | 50 | 50/1250 | 2.99E-07 | 2.64E-06 | 2.1315976 |
| GO_BP | GO:0031667~response to nutrient levels | 66 | 66/1250 | 3.01E-07 | 2.64E-06 | 1.905879 |
| GO_BP | GO:0031532~actin cytoskeleton reorganization | 23 | 23/1250 | 3.12E-07 | 2.72E-06 | 3.2813941 |
| GO_BP | GO:0045667~regulation of osteoblast differentiation | 25 | 25/1250 | 4.23E-07 | 3.59E-06 | 3.0528814 |
| GO_BP | GO:0007565~female pregnancy | 34 | 34/1250 | 4.29E-07 | 3.64E-06 | 2.5384788 |
| GO_BP | GO:0010324~membrane invagination | 18 | 18/1250 | 4.41E-07 | 3.72E-06 | 3.8712358 |
| GO_BP | GO:1901861~regulation of muscle tissue development | 29 | 29/1250 | 4.72E-07 | 3.93E-06 | 2.7674066 |
| GO_BP | GO:0031341~regulation of cell killing | 22 | 22/1250 | 5.00E-07 | 4.12E-06 | 3.3022 |
| GO_BP | GO:0002576~platelet degranulation | 26 | 26/1250 | 5.07E-07 | 4.17E-06 | 2.9499969 |
| GO_BP | GO:0060021~roof of mouth development | 21 | 21/1250 | 5.39E-07 | 4.42E-06 | 3.400018 |
| GO_BP | GO:0097306~cellular response to alcohol | 21 | 21/1250 | 5.39E-07 | 4.42E-06 | 3.400018 |
| GO_BP | GO:0036465~synaptic vesicle recycling | 18 | 18/1250 | 5.61E-07 | 4.58E-06 | 3.8143059 |
| GO_BP | GO:0150063~visual system development | 52 | 52/1250 | 5.75E-07 | 4.69E-06 | 2.0528745 |
| GO_BP | GO:0044706~multi-multicellular organism process | 37 | 37/1250 | 6.33E-07 | 5.14E-06 | 2.3908305 |
| GO_BP | GO:0007009~plasma membrane organization | 23 | 23/1250 | 6.53E-07 | 5.28E-06 | 3.1563886 |
| GO_BP | GO:0071496~cellular response to external stimulus | 49 | 49/1250 | 7.22E-07 | 5.76E-06 | 2.0889657 |
| GO_BP | GO:0032386~regulation of intracellular transport | 57 | 57/1250 | 8.40E-07 | 6.60E-06 | 1.9555886 |
| GO_BP | GO:0070542~response to fatty acid | 20 | 20/1250 | 8.56E-07 | 6.67E-06 | 3.4308571 |
| GO_BP | GO:0016572~histone phosphorylation | 13 | 13/1250 | 8.56E-07 | 6.67E-06 | 4.9296 |
| GO_BP | GO:0010463~mesenchymal cell proliferation | 14 | 14/1250 | 8.92E-07 | 6.93E-06 | 4.5848727 |
| GO_BP | GO:0002831~regulation of response to biotic stimulus | 27 | 27/1250 | 9.21E-07 | 7.12E-06 | 2.7989871 |
| GO_BP | GO:0001505~regulation of neurotransmitter levels | 50 | 50/1250 | 9.48E-07 | 7.32E-06 | 2.0526496 |
| GO_BP | GO:0042107~cytokine metabolic process | 25 | 25/1250 | 9.62E-07 | 7.39E-06 | 2.9287805 |
| GO_BP | GO:0050807~regulation of synapse organization | 36 | 36/1250 | 1.00E-06 | 7.70E-06 | 2.379567 |
| GO_BP | GO:0009896~positive regulation of catabolic process | 57 | 57/1250 | 1.06E-06 | 8.10E-06 | 1.9417191 |
| GO_BP | GO:0001570~vasculogenesis | 19 | 19/1250 | 1.10E-06 | 8.40E-06 | 3.5100308 |
| GO_BP | GO:0032535~regulation of cellular component size | 51 | 51/1250 | 1.56E-06 | 1.16E-05 | 2.002424 |
| GO_BP | GO:0034103~regulation of tissue remodeling | 19 | 19/1250 | 1.67E-06 | 1.22E-05 | 3.42228 |
| GO_BP | GO:0099024~plasma membrane invagination | 16 | 16/1250 | 1.67E-06 | 1.23E-05 | 3.9076881 |
| GO_BP | GO:1990778~protein localization to cell periphery | 45 | 45/1250 | 1.79E-06 | 1.30E-05 | 2.0984854 |
| GO_BP | GO:0099003~vesicle-mediated transport in synapse | 34 | 34/1250 | 1.80E-06 | 1.30E-05 | 2.3898849 |
| GO_BP | GO:0019730~antimicrobial humoral response | 24 | 24/1250 | 1.82E-06 | 1.32E-05 | 2.9061378 |
| GO_BP | GO:0031623~receptor internalization | 23 | 23/1250 | 1.82E-06 | 1.32E-05 | 2.985773 |
| GO_BP | GO:0003018~vascular process in circulatory system | 30 | 30/1250 | 1.94E-06 | 1.40E-05 | 2.5428706 |
| GO_BP | GO:0042271~susceptibility to natural killer cell mediated cytotoxicity | 7 | 7/1250 | 1.96E-06 | 1.40E-05 | 9.1697455 |
| GO_BP | GO:0061614~pri-miRNA transcription by RNA polymerase II | 14 | 14/1250 | 2.18E-06 | 1.52E-05 | 4.2922213 |
| GO_BP | GO:0045730~respiratory burst | 12 | 12/1250 | 2.21E-06 | 1.54E-05 | 4.9404343 |
| GO_BP | GO:0060402~calcium ion transport into cytosol | 28 | 28/1250 | 2.64E-06 | 1.82E-05 | 2.6030245 |
| GO_BP | GO:0048678~response to axon injury | 18 | 18/1250 | 2.65E-06 | 1.83E-05 | 3.458304 |
| GO_BP | GO:0031644~regulation of neurological system process | 26 | 26/1250 | 2.65E-06 | 1.83E-05 | 2.7148522 |
| GO_BP | GO:0071398~cellular response to fatty acid | 15 | 15/1250 | 3.25E-06 | 2.18E-05 | 3.9298909 |
| GO_BP | GO:0007589~body fluid secretion | 20 | 20/1250 | 3.91E-06 | 2.59E-05 | 3.1325217 |
| GO_BP | GO:0098911~regulation of ventricular cardiac muscle cell action potential | 8 | 8/1250 | 4.09E-06 | 2.70E-05 | 7.2048 |
| GO_BP | GO:0000768~syncytium formation by plasma membrane fusion | 15 | 15/1250 | 4.16E-06 | 2.73E-05 | 3.8597143 |
| GO_BP | GO:0140253~cell-cell fusion | 15 | 15/1250 | 4.16E-06 | 2.73E-05 | 3.8597143 |
| GO_BP | GO:1903522~regulation of blood circulation | 42 | 42/1250 | 4.52E-06 | 2.95E-05 | 2.0869076 |
| GO_BP | GO:0042133~neurotransmitter metabolic process | 27 | 27/1250 | 4.83E-06 | 3.13E-05 | 2.576551 |
| GO_BP | GO:0044783~G1 DNA damage checkpoint | 16 | 16/1250 | 5.35E-06 | 3.42E-05 | 3.6024 |
| GO_BP | GO:0030010~establishment of cell polarity | 25 | 25/1250 | 5.65E-06 | 3.60E-05 | 2.6684444 |
| GO_BP | GO:0098693~regulation of synaptic vesicle cycle | 22 | 22/1250 | 6.53E-06 | 4.12E-05 | 2.8559568 |
| GO_BP | GO:1903317~regulation of protein maturation | 23 | 23/1250 | 7.31E-06 | 4.56E-05 | 2.76184 |
| GO_BP | GO:0150076~neuroinflammatory response | 17 | 17/1250 | 7.72E-06 | 4.80E-05 | 3.3556603 |
| GO_BP | GO:0032956~regulation of actin cytoskeleton organization | 46 | 46/1250 | 8.12E-06 | 4.99E-05 | 1.966889 |
| GO_BP | GO:0042267~natural killer cell mediated cytotoxicity | 15 | 15/1250 | 8.38E-06 | 5.12E-05 | 3.6634576 |
| GO_BP | GO:0042035~regulation of cytokine biosynthetic process | 22 | 22/1250 | 8.83E-06 | 5.37E-05 | 2.8054089 |
| GO_BP | GO:0051893~regulation of focal adhesion assembly | 15 | 15/1250 | 1.05E-05 | 6.27E-05 | 3.6024 |
| GO_BP | GO:0031331~positive regulation of cellular catabolic process | 48 | 48/1250 | 1.06E-05 | 6.36E-05 | 1.9159579 |
| GO_BP | GO:0050880~regulation of blood vessel size | 25 | 25/1250 | 1.10E-05 | 6.53E-05 | 2.5731429 |
| GO_BP | GO:0097746~regulation of blood vessel diameter | 25 | 25/1250 | 1.10E-05 | 6.53E-05 | 2.5731429 |
| GO_BP | GO:0110111~negative regulation of animal organ morphogenesis | 11 | 11/1250 | 1.11E-05 | 6.60E-05 | 4.6619294 |
| GO_BP | GO:0060973~cell migration involved in heart development | 8 | 8/1250 | 1.23E-05 | 7.22E-05 | 6.4042667 |
| GO_BP | GO:0033627~cell adhesion mediated by integrin | 16 | 16/1250 | 1.24E-05 | 7.23E-05 | 3.3904941 |
| GO_BP | GO:0002269~leukocyte activation involved in inflammatory response | 13 | 13/1250 | 1.24E-05 | 7.23E-05 | 3.985634 |
| GO_BP | GO:0007595~lactation | 13 | 13/1250 | 1.24E-05 | 7.23E-05 | 3.985634 |
| GO_BP | GO:0140238~presynaptic endocytosis | 14 | 14/1250 | 1.64E-05 | 9.34E-05 | 3.6678982 |
| GO_BP | GO:1901655~cellular response to ketone | 19 | 19/1250 | 1.74E-05 | 9.86E-05 | 2.9438968 |
| GO_BP | GO:0062009~secondary palate development | 9 | 9/1250 | 1.83E-05 | 0.0001029 | 5.4036 |
| GO_BP | GO:0097479~synaptic vesicle localization | 27 | 27/1250 | 1.84E-05 | 0.0001035 | 2.4016 |
| GO_BP | GO:0098581~detection of external biotic stimulus | 8 | 8/1250 | 1.99E-05 | 0.0001113 | 6.0672 |
| GO_BP | GO:0050804~modulation of chemical synaptic transmission | 54 | 54/1250 | 2.26E-05 | 0.0001248 | 1.7887779 |
| GO_BP | GO:0099177~regulation of trans-synaptic signaling | 54 | 54/1250 | 2.41E-05 | 0.0001321 | 1.7846752 |
| GO_BP | GO:0061900~glial cell activation | 14 | 14/1250 | 2.55E-05 | 0.0001393 | 3.5392 |
| GO_BP | GO:0010038~response to metal ion | 47 | 47/1250 | 2.61E-05 | 0.0001428 | 1.8657058 |
| GO_BP | GO:1905314~semi-lunar valve development | 11 | 11/1250 | 2.74E-05 | 0.0001478 | 4.2839351 |
| GO_BP | GO:1901653~cellular response to peptide | 49 | 49/1250 | 2.80E-05 | 0.0001506 | 1.8339491 |
| GO_BP | GO:0007006~mitochondrial membrane organization | 23 | 23/1250 | 2.84E-05 | 0.0001526 | 2.5493908 |
| GO_BP | GO:0098901~regulation of cardiac muscle cell action potential | 10 | 10/1250 | 2.88E-05 | 0.0001543 | 4.6482581 |
| GO_BP | GO:0051668~localization within membrane | 25 | 25/1250 | 2.94E-05 | 0.0001572 | 2.4340541 |
| GO_BP | GO:0051767~nitric-oxide synthase biosynthetic process | 8 | 8/1250 | 3.12E-05 | 0.0001653 | 5.76384 |
| GO_BP | GO:0051769~regulation of nitric-oxide synthase biosynthetic process | 8 | 8/1250 | 3.12E-05 | 0.0001653 | 5.76384 |
| GO_BP | GO:0031102~neuron projection regeneration | 14 | 14/1250 | 3.15E-05 | 0.0001659 | 3.4781793 |
| GO_BP | GO:0000281~mitotic cytokinesis | 16 | 16/1250 | 3.19E-05 | 0.000168 | 3.1582685 |
| GO_BP | GO:0071333~cellular response to glucose stimulus | 23 | 23/1250 | 3.22E-05 | 0.0001694 | 2.5299298 |
| GO_BP | GO:1905517~macrophage migration | 13 | 13/1250 | 3.23E-05 | 0.0001694 | 3.6730353 |
| GO_BP | GO:0048843~negative regulation of axon extension involved in axon guidance | 9 | 9/1250 | 3.85E-05 | 0.0001992 | 4.9879385 |
| GO_BP | GO:0097755~positive regulation of blood vessel diameter | 14 | 14/1250 | 3.86E-05 | 0.0001999 | 3.4192271 |
| GO_BP | GO:0071402~cellular response to lipoprotein particle stimulus | 10 | 10/1250 | 3.93E-05 | 0.0002025 | 4.503 |
| GO_BP | GO:0009266~response to temperature stimulus | 34 | 34/1250 | 4.34E-05 | 0.0002212 | 2.0672 |
| GO_BP | GO:1905207~regulation of cardiocyte differentiation | 14 | 14/1250 | 4.72E-05 | 0.0002379 | 3.36224 |
| GO_BP | GO:0097067~cellular response to thyroid hormone stimulus | 7 | 7/1250 | 4.99E-05 | 0.0002509 | 6.3042 |
| GO_BP | GO:0003014~renal system process | 21 | 21/1250 | 5.01E-05 | 0.0002512 | 2.5863385 |
| GO_BP | GO:0032409~regulation of transporter activity | 38 | 38/1250 | 5.08E-05 | 0.0002541 | 1.9625978 |
| GO_BP | GO:0042593~glucose homeostasis | 34 | 34/1250 | 5.15E-05 | 0.0002578 | 2.0499013 |
| GO_BP | GO:0010464~regulation of mesenchymal cell proliferation | 10 | 10/1250 | 5.29E-05 | 0.0002637 | 4.3665455 |
| GO_BP | GO:0071383~cellular response to steroid hormone stimulus | 35 | 35/1250 | 5.62E-05 | 0.0002792 | 2.017344 |
| GO_BP | GO:0007088~regulation of mitotic nuclear division | 26 | 26/1250 | 6.29E-05 | 0.0003091 | 2.2844488 |
| GO_BP | GO:0035821~modification of morphology or physiology of other organism | 26 | 26/1250 | 6.29E-05 | 0.0003091 | 2.2844488 |
| GO_BP | GO:0050886~endocrine process | 17 | 17/1250 | 6.32E-05 | 0.0003102 | 2.88192 |
| GO_BP | GO:1902337~regulation of apoptotic process involved in morphogenesis | 6 | 6/1250 | 7.09E-05 | 0.0003428 | 7.2048 |
| GO_BP | GO:1904748~regulation of apoptotic process involved in development | 6 | 6/1250 | 7.09E-05 | 0.0003428 | 7.2048 |
| GO_BP | GO:0042306~regulation of protein import into nucleus | 13 | 13/1250 | 7.58E-05 | 0.0003639 | 3.4059055 |
| GO_BP | GO:0051302~regulation of cell division | 26 | 26/1250 | 9.49E-05 | 0.0004472 | 2.2300571 |
| GO_BP | GO:0001678~cellular glucose homeostasis | 24 | 24/1250 | 0.0001024 | 0.0004778 | 2.305536 |
| GO_BP | GO:0010256~endomembrane system organization | 52 | 52/1250 | 0.0001028 | 0.0004797 | 1.7107288 |
| GO_BP | GO:0071214~cellular response to abiotic stimulus | 42 | 42/1250 | 0.000109 | 0.0005049 | 1.8284085 |
| GO_BP | GO:0104004~cellular response to environmental stimulus | 42 | 42/1250 | 0.000109 | 0.0005049 | 1.8284085 |
| GO_BP | GO:0097480~establishment of synaptic vesicle localization | 24 | 24/1250 | 0.0001138 | 0.000526 | 2.2902676 |
| GO_BP | GO:2001021~negative regulation of response to DNA damage stimulus | 16 | 16/1250 | 0.0001202 | 0.0005497 | 2.8463407 |
| GO_BP | GO:0007183~SMAD protein complex assembly | 6 | 6/1250 | 0.0001239 | 0.0005597 | 6.6505846 |
| GO_BP | GO:0099173~postsynapse organization | 25 | 25/1250 | 0.0001344 | 0.0006035 | 2.2237037 |
| GO_BP | GO:0035306~positive regulation of dephosphorylation | 13 | 13/1250 | 0.0001357 | 0.0006076 | 3.2297379 |
| GO_BP | GO:0050690~regulation of defense response to virus by virus | 9 | 9/1250 | 0.0001367 | 0.0006114 | 4.32288 |
| GO_BP | GO:0055094~response to lipoprotein particle | 9 | 9/1250 | 0.0001367 | 0.0006114 | 4.32288 |
| GO_BP | GO:0061844~antimicrobial humoral immune response mediated by antimicrobial peptide | 15 | 15/1250 | 0.0001434 | 0.0006384 | 2.9208649 |
| GO_BP | GO:0031647~regulation of protein stability | 37 | 37/1250 | 0.0001492 | 0.000663 | 1.8839406 |
| GO_BP | GO:1905897~regulation of response to endoplasmic reticulum stress | 16 | 16/1250 | 0.0001622 | 0.0007167 | 2.7777542 |
| GO_BP | GO:1902170~cellular response to reactive nitrogen species | 7 | 7/1250 | 0.0001829 | 0.0007977 | 5.3088 |
| GO_BP | GO:0045927~positive regulation of growth | 35 | 35/1250 | 0.0001964 | 0.0008471 | 1.896 |
| GO_BP | GO:0031668~cellular response to extracellular stimulus | 35 | 35/1250 | 0.0002113 | 0.0009059 | 1.8888989 |
| GO_BP | GO:0014074~response to purine-containing compound | 23 | 23/1250 | 0.0002205 | 0.0009385 | 2.2393297 |
| GO_BP | GO:0007416~synapse assembly | 26 | 26/1250 | 0.0002253 | 0.0009584 | 2.1166644 |
| GO_BP | GO:0019216~regulation of lipid metabolic process | 48 | 48/1250 | 0.0002453 | 0.0010344 | 1.6911022 |
| GO_BP | GO:0016050~vesicle organization | 40 | 40/1250 | 0.0002465 | 0.0010387 | 1.7900124 |
| GO_BP | GO:0150077~regulation of neuroinflammatory response | 10 | 10/1250 | 0.0002479 | 0.0010425 | 3.6947692 |
| GO_BP | GO:0051341~regulation of oxidoreductase activity | 18 | 18/1250 | 0.0002704 | 0.001126 | 2.4939692 |
| GO_BP | GO:0001756~somitogenesis | 14 | 14/1250 | 0.0002743 | 0.0011399 | 2.88192 |
| GO_BP | GO:0002532~production of molecular mediator involved in inflammatory response | 14 | 14/1250 | 0.0002743 | 0.0011399 | 2.88192 |
| GO_BP | GO:0009410~response to xenobiotic stimulus | 37 | 37/1250 | 0.0003 | 0.0012345 | 1.8196423 |
| GO_BP | GO:0043551~regulation of phosphatidylinositol 3-kinase activity | 12 | 12/1250 | 0.0003183 | 0.0012979 | 3.1439127 |
| GO_BP | GO:0046683~response to organophosphorus | 21 | 21/1250 | 0.000326 | 0.0013196 | 2.2752 |
| GO_BP | GO:0140131~positive regulation of lymphocyte chemotaxis | 7 | 7/1250 | 0.0003732 | 0.0014911 | 4.8032 |
| GO_BP | GO:0061053~somite development | 16 | 16/1250 | 0.0003739 | 0.0014931 | 2.5904899 |
| GO_BP | GO:0140014~mitotic nuclear division | 34 | 34/1250 | 0.0003862 | 0.0015279 | 1.8487789 |
| GO_BP | GO:0031128~developmental induction | 9 | 9/1250 | 0.0003896 | 0.0015382 | 3.8143059 |
| GO_BP | GO:0061351~neural precursor cell proliferation | 22 | 22/1250 | 0.0004645 | 0.0018194 | 2.1713096 |
| GO_BP | GO:0034698~response to gonadotropin | 8 | 8/1250 | 0.0004666 | 0.001823 | 4.1170286 |
| GO_BP | GO:0030214~hyaluronan catabolic process | 6 | 6/1250 | 0.0004822 | 0.0018691 | 5.4036 |
| GO_BP | GO:1905153~regulation of membrane invagination | 6 | 6/1250 | 0.0004822 | 0.0018691 | 5.4036 |
| GO_BP | GO:1905331~negative regulation of morphogenesis of an epithelium | 6 | 6/1250 | 0.0004822 | 0.0018691 | 5.4036 |
| GO_BP | GO:1990000~amyloid fibril formation | 6 | 6/1250 | 0.0004822 | 0.0018691 | 5.4036 |
| GO_BP | GO:0022600~digestive system process | 17 | 17/1250 | 0.0004913 | 0.0018966 | 2.449632 |
| GO_BP | GO:0034405~response to fluid shear stress | 9 | 9/1250 | 0.0004925 | 0.0018966 | 3.7053257 |
| GO_BP | GO:0086005~ventricular cardiac muscle cell action potential | 9 | 9/1250 | 0.0004925 | 0.0018966 | 3.7053257 |
| GO_BP | GO:1905209~positive regulation of cardiocyte differentiation | 9 | 9/1250 | 0.0004925 | 0.0018966 | 3.7053257 |
| GO_BP | GO:0010869~regulation of receptor biosynthetic process | 7 | 7/1250 | 0.0005147 | 0.0019624 | 4.5848727 |
| GO_BP | GO:0090594~inflammatory response to wounding | 5 | 5/1250 | 0.0005178 | 0.0019624 | 6.5498182 |
| GO_BP | GO:1904888~cranial skeletal system development | 13 | 13/1250 | 0.0005241 | 0.0019784 | 2.8382545 |
| GO_BP | GO:0006970~response to osmotic stress | 15 | 15/1250 | 0.0005319 | 0.0020053 | 2.6041446 |
| GO_BP | GO:0110020~regulation of actomyosin structure organization | 16 | 16/1250 | 0.000549 | 0.0020686 | 2.5060174 |
| GO_BP | GO:1900407~regulation of cellular response to oxidative stress | 14 | 14/1250 | 0.0005753 | 0.0021635 | 2.689792 |
| GO_BP | GO:0038084~vascular endothelial growth factor signaling pathway | 10 | 10/1250 | 0.0005801 | 0.0021773 | 3.3510698 |
| GO_BP | GO:0050974~detection of mechanical stimulus involved in sensory perception | 8 | 8/1250 | 0.0006056 | 0.0022617 | 3.9750621 |
| GO_BP | GO:0009145~purine nucleoside triphosphate biosynthetic process | 24 | 24/1250 | 0.0006362 | 0.0023613 | 2.0463337 |
| GO_BP | GO:0033599~regulation of mammary gland epithelial cell proliferation | 6 | 6/1250 | 0.0007017 | 0.0025754 | 5.0857412 |
| GO_BP | GO:0072673~lamellipodium morphogenesis | 6 | 6/1250 | 0.0007017 | 0.0025754 | 5.0857412 |
| GO_BP | GO:0030212~hyaluronan metabolic process | 9 | 9/1250 | 0.0007653 | 0.0027831 | 3.5050378 |
| GO_BP | GO:1905898~positive regulation of response to endoplasmic reticulum stress | 9 | 9/1250 | 0.0007653 | 0.0027831 | 3.5050378 |
| GO_BP | GO:0051260~protein homooligomerization | 41 | 41/1250 | 0.0007821 | 0.0028337 | 1.6783909 |
| GO_BP | GO:0097327~response to antineoplastic agent | 16 | 16/1250 | 0.00079 | 0.0028606 | 2.42688 |
| GO_BP | GO:0007077~mitotic nuclear envelope disassembly | 5 | 5/1250 | 0.0008371 | 0.003011 | 6.004 |
| GO_BP | GO:0051963~regulation of synapse assembly | 17 | 17/1250 | 0.0009738 | 0.003432 | 2.3109736 |
| GO_BP | GO:1905276~regulation of epithelial tube formation | 6 | 6/1250 | 0.0009912 | 0.0034623 | 4.8032 |
| GO_BP | GO:0022602~ovulation cycle process | 10 | 10/1250 | 0.00102 | 0.0035484 | 3.1325217 |
| GO_BP | GO:0030048~actin filament-based movement | 20 | 20/1250 | 0.0010239 | 0.0035601 | 2.1347556 |
| GO_BP | GO:0099175~regulation of postsynapse organization | 16 | 16/1250 | 0.0011157 | 0.0038612 | 2.3525878 |
| GO_BP | GO:0035418~protein localization to synapse | 12 | 12/1250 | 0.0011583 | 0.0039877 | 2.7446857 |
| GO_BP | GO:0120192~tight junction assembly | 11 | 11/1250 | 0.0012106 | 0.0041535 | 2.88192 |
| GO_BP | GO:0048066~developmental pigmentation | 10 | 10/1250 | 0.0012163 | 0.0041535 | 3.0658723 |
| GO_BP | GO:0043304~regulation of mast cell degranulation | 8 | 8/1250 | 0.001232 | 0.0041928 | 3.6024 |
| GO_BP | GO:0010506~regulation of autophagy | 38 | 38/1250 | 0.0012459 | 0.0042353 | 1.6745101 |
| GO_BP | GO:0048511~rhythmic process | 35 | 35/1250 | 0.0012529 | 0.0042542 | 1.7154286 |
| GO_BP | GO:0097756~negative regulation of blood vessel diameter | 14 | 14/1250 | 0.0012687 | 0.0043027 | 2.4905481 |
| GO_BP | GO:0016079~synaptic vesicle exocytosis | 18 | 18/1250 | 0.0012731 | 0.0043104 | 2.1980746 |
| GO_BP | GO:1905155~positive regulation of membrane invagination | 5 | 5/1250 | 0.0012829 | 0.0043264 | 5.5421538 |
| GO_BP | GO:0032963~collagen metabolic process | 17 | 17/1250 | 0.0013381 | 0.0045047 | 2.2473688 |
| GO_BP | GO:0019835~cytolysis | 9 | 9/1250 | 0.0013939 | 0.0046505 | 3.24216 |
| GO_BP | GO:0061077~chaperone-mediated protein folding | 11 | 11/1250 | 0.0014137 | 0.0047006 | 2.8304571 |
| GO_BP | GO:0071825~protein-lipid complex subunit organization | 10 | 10/1250 | 0.0014423 | 0.0047799 | 3.002 |
| GO_BP | GO:0033006~regulation of mast cell activation involved in immune response | 8 | 8/1250 | 0.0015286 | 0.0050433 | 3.4932364 |
| GO_BP | GO:0014823~response to activity | 12 | 12/1250 | 0.0015384 | 0.0050699 | 2.6602338 |
| GO_BP | GO:0043403~skeletal muscle tissue regeneration | 9 | 9/1250 | 0.0016776 | 0.0054862 | 3.1630829 |
| GO_BP | GO:0003044~regulation of systemic arterial blood pressure mediated by a chemical signal | 10 | 10/1250 | 0.0017013 | 0.0055516 | 2.9407347 |
| GO_BP | GO:0003254~regulation of membrane depolarization | 10 | 10/1250 | 0.0017013 | 0.0055516 | 2.9407347 |
| GO_BP | GO:1902882~regulation of response to oxidative stress | 14 | 14/1250 | 0.0018193 | 0.0059039 | 2.4016 |
| GO_BP | GO:0001771~immunological synapse formation | 5 | 5/1250 | 0.0018823 | 0.0060556 | 5.1462857 |
| GO_BP | GO:0045198~establishment of epithelial cell apical/basal polarity | 5 | 5/1250 | 0.0018823 | 0.0060556 | 5.1462857 |
| GO_BP | GO:0086069~bundle of His cell to Purkinje myocyte communication | 5 | 5/1250 | 0.0018823 | 0.0060556 | 5.1462857 |
| GO_BP | GO:0062013~positive regulation of small molecule metabolic process | 21 | 21/1250 | 0.0019096 | 0.0061302 | 1.9908 |
| GO_BP | GO:0009895~negative regulation of catabolic process | 35 | 35/1250 | 0.0019872 | 0.006362 | 1.6699868 |
| GO_BP | GO:0030301~cholesterol transport | 15 | 15/1250 | 0.0019889 | 0.0063642 | 2.2994043 |
| GO_BP | GO:0007611~learning or memory | 31 | 31/1250 | 0.001994 | 0.0063769 | 1.731386 |
| GO_BP | GO:0031641~regulation of myelination | 9 | 9/1250 | 0.0020058 | 0.0064009 | 3.0877714 |
| GO_BP | GO:0106106~cold-induced thermogenesis | 20 | 20/1250 | 0.0020867 | 0.0066414 | 2.0153287 |
| GO_BP | GO:0120161~regulation of cold-induced thermogenesis | 20 | 20/1250 | 0.0020867 | 0.0066414 | 2.0153287 |
| GO_BP | GO:0120193~tight junction organization | 11 | 11/1250 | 0.002196 | 0.0069742 | 2.6865356 |
| GO_BP | GO:1905332~positive regulation of morphogenesis of an epithelium | 8 | 8/1250 | 0.0022899 | 0.0072376 | 3.2936229 |
| GO_BP | GO:0006692~prostanoid metabolic process | 9 | 9/1250 | 0.0023832 | 0.0074689 | 3.0159628 |
| GO_BP | GO:0006693~prostaglandin metabolic process | 9 | 9/1250 | 0.0023832 | 0.0074689 | 3.0159628 |
| GO_BP | GO:0036499~PERK-mediated unfolded protein response | 6 | 6/1250 | 0.0024205 | 0.0075419 | 4.1170286 |
| GO_BP | GO:0034369~plasma lipoprotein particle remodeling | 7 | 7/1250 | 0.0024767 | 0.0076849 | 3.6024 |
| GO_BP | GO:0044406~adhesion of symbiont to host | 5 | 5/1250 | 0.0026634 | 0.0082084 | 4.8032 |
| GO_BP | GO:0120162~positive regulation of cold-induced thermogenesis | 15 | 15/1250 | 0.0027268 | 0.0083995 | 2.2282887 |
| GO_BP | GO:0106027~neuron projection organization | 14 | 14/1250 | 0.0028487 | 0.0087119 | 2.2924364 |
| GO_BP | GO:0072655~establishment of protein localization to mitochondrion | 19 | 19/1250 | 0.0029316 | 0.0089514 | 1.9984117 |
| GO_BP | GO:0043473~pigmentation | 15 | 15/1250 | 0.0030179 | 0.0092009 | 2.205551 |
| GO_BP | GO:0070723~response to cholesterol | 7 | 7/1250 | 0.0030721 | 0.0093325 | 3.4781793 |
| GO_BP | GO:0036297~interstrand cross-link repair | 10 | 10/1250 | 0.003138 | 0.009488 | 2.7187925 |
| GO_BP | GO:0140029~exocytic process | 13 | 13/1250 | 0.003282 | 0.0098942 | 2.34156 |
| GO_BP | GO:1905954~positive regulation of lipid localization | 13 | 13/1250 | 0.003282 | 0.0098942 | 2.34156 |
| GO_BP | GO:0030810~positive regulation of nucleotide biosynthetic process | 9 | 9/1250 | 0.003306 | 0.0099263 | 2.88192 |
| GO_BP | GO:1900373~positive regulation of purine nucleotide biosynthetic process | 9 | 9/1250 | 0.003306 | 0.0099263 | 2.88192 |
| GO_BP | GO:0098727~maintenance of cell number | 21 | 21/1250 | 0.0033151 | 0.0099296 | 1.9031547 |
| GO_BP | GO:1905521~regulation of macrophage migration | 8 | 8/1250 | 0.0033204 | 0.0099296 | 3.1155892 |
| GO_CC | GO:0062023~collagen-containing extracellular matrix | 96 | 96/1257 | 7.37E-29 | 4.58E-26 | 3.57347 |
| GO_CC | GO:0009897~external side of plasma membrane | 75 | 75/1257 | 6.97E-23 | 2.17E-20 | 3.589423 |
| GO_CC | GO:0005911~cell-cell junction | 92 | 92/1257 | 4.14E-22 | 8.58E-20 | 3.0216842 |
| GO_CC | GO:0005924~cell-substrate adherens junction | 77 | 77/1257 | 4.15E-17 | 6.45E-15 | 2.8451456 |
| GO_CC | GO:0030055~cell-substrate junction | 77 | 77/1257 | 7.35E-17 | 9.15E-15 | 2.8175228 |
| GO_CC | GO:0031983~vesicle lumen | 67 | 67/1257 | 4.39E-16 | 3.90E-14 | 2.9795388 |
| GO_CC | GO:0060205~cytoplasmic vesicle lumen | 66 | 66/1257 | 1.36E-15 | 1.06E-13 | 2.9437517 |
| GO_CC | GO:0031252~cell leading edge | 70 | 70/1257 | 9.91E-14 | 5.60E-12 | 2.6121049 |
| GO_CC | GO:0043235~receptor complex | 68 | 68/1257 | 3.09E-13 | 1.60E-11 | 2.5952892 |
| GO_CC | GO:0005913~cell-cell adherens junction | 33 | 33/1257 | 5.25E-13 | 2.41E-11 | 4.2520858 |
| GO_CC | GO:0000502~proteasome complex | 24 | 24/1257 | 5.42E-13 | 2.41E-11 | 5.7430769 |
| GO_CC | GO:1905369~endopeptidase complex | 24 | 24/1257 | 8.14E-13 | 3.37E-11 | 5.6533413 |
| GO_CC | GO:0098589~membrane region | 59 | 59/1257 | 2.03E-12 | 7.88E-11 | 2.7035229 |
| GO_CC | GO:0005788~endoplasmic reticulum lumen | 56 | 56/1257 | 4.19E-12 | 1.53E-10 | 2.741014 |
| GO_CC | GO:0044420~extracellular matrix component | 20 | 20/1257 | 2.50E-11 | 6.95E-10 | 5.9119909 |
| GO_CC | GO:0030666~endocytic vesicle membrane | 36 | 36/1257 | 1.36E-10 | 3.52E-09 | 3.309273 |
| GO_CC | GO:1905368~peptidase complex | 24 | 24/1257 | 9.78E-10 | 2.10E-08 | 4.2071377 |
| GO_CC | GO:0045177~apical part of cell | 59 | 59/1257 | 1.48E-09 | 3.07E-08 | 2.304298 |
| GO_CC | GO:0019898~extrinsic component of membrane | 49 | 49/1257 | 1.80E-09 | 3.61E-08 | 2.5211715 |
| GO_CC | GO:0016324~apical plasma membrane | 50 | 50/1257 | 1.14E-08 | 2.15E-07 | 2.3629431 |
| GO_CC | GO:0005604~basement membrane | 23 | 23/1257 | 3.37E-08 | 6.00E-07 | 3.688705 |
| GO_CC | GO:0098978~glutamatergic synapse | 50 | 50/1257 | 2.11E-07 | 3.45E-06 | 2.1598248 |
| GO_CC | GO:1902911~protein kinase complex | 23 | 23/1257 | 5.06E-07 | 7.50E-06 | 3.2105395 |
| GO_CC | GO:0005581~collagen trimer | 20 | 20/1257 | 7.70E-07 | 1.09E-05 | 3.4656498 |
| GO_CC | GO:0098793~presynapse | 62 | 62/1257 | 8.66E-07 | 1.20E-05 | 1.8959143 |
| GO_CC | GO:0005902~microvillus | 19 | 19/1257 | 1.55E-06 | 1.82E-05 | 3.4510356 |
| GO_CC | GO:0098636~protein complex involved in cell adhesion | 11 | 11/1257 | 7.23E-06 | 8.04E-05 | 4.8773925 |
| GO_CC | GO:0005884~actin filament | 20 | 20/1257 | 3.31E-05 | 0.0003116 | 2.741014 |
| GO_CC | GO:0005938~cell cortex | 40 | 40/1257 | 3.57E-05 | 0.0003216 | 1.9578671 |
| GO_CC | GO:0031225~anchored component of membrane | 26 | 26/1257 | 5.02E-05 | 0.0004394 | 2.3193195 |
| GO_CC | GO:0031594~neuromuscular junction | 15 | 15/1257 | 0.0001194 | 0.0009399 | 2.9754428 |
| GO_CC | GO:0005769~early endosome | 42 | 42/1257 | 0.0001253 | 0.0009746 | 1.8194662 |
| GO_CC | GO:1990391~DNA repair complex | 10 | 10/1257 | 0.0001356 | 0.0010287 | 3.967257 |
| GO_CC | GO:0031588~nucleotide-activated protein kinase complex | 6 | 6/1257 | 0.000159 | 0.0011919 | 6.4609615 |
| GO_CC | GO:0070938~contractile ring | 5 | 5/1257 | 0.0002422 | 0.0016556 | 7.5377884 |
| GO_CC | GO:0033267~axon part | 44 | 44/1257 | 0.000252 | 0.0017035 | 1.7364539 |
| GO_CC | GO:0044798~nuclear transcription factor complex | 27 | 27/1257 | 0.0003121 | 0.0020649 | 2.04543 |
| GO_CC | GO:0071437~invadopodium | 6 | 6/1257 | 0.000378 | 0.0024488 | 5.6533413 |
| GO_CC | GO:0045178~basal part of cell | 11 | 11/1257 | 0.0004249 | 0.0026698 | 3.251595 |
| GO_CC | GO:0010008~endosome membrane | 50 | 50/1257 | 0.000862 | 0.0051555 | 1.583569 |
| GO_CC | GO:0001533~cornified envelope | 12 | 12/1257 | 0.0010441 | 0.0060216 | 2.7831834 |
| GO_CC | GO:0099243~extrinsic component of synaptic membrane | 5 | 5/1257 | 0.0010456 | 0.0060216 | 5.7982988 |
| GO_CC | GO:0072562~blood microparticle | 17 | 17/1257 | 0.0013617 | 0.0077092 | 2.2481123 |
| GO_CC | GO:0043083~synaptic cleft | 5 | 5/1257 | 0.0015381 | 0.0085417 | 5.3841346 |
| GO_CC | GO:0032153~cell division site | 12 | 12/1257 | 0.0015716 | 0.0086509 | 2.6603959 |
| GO_MF | GO:0050839~cell adhesion molecule binding | 106 | 106/1227 | 2.30E-25 | 2.17E-22 | 3.005905 |
| GO_MF | GO:0005178~integrin binding | 43 | 43/1227 | 4.52E-18 | 2.13E-15 | 4.5911375 |
| GO_MF | GO:0019955~cytokine binding | 41 | 41/1227 | 5.36E-17 | 1.69E-14 | 4.5143961 |
| GO_MF | GO:0005539~glycosaminoglycan binding | 56 | 56/1227 | 8.97E-17 | 1.86E-14 | 3.4616166 |
| GO_MF | GO:0005126~cytokine receptor binding | 64 | 64/1227 | 9.87E-17 | 1.86E-14 | 3.1428515 |
| GO_MF | GO:0048018~receptor ligand activity | 80 | 80/1227 | 6.93E-13 | 9.34E-11 | 2.3343643 |
| GO_MF | GO:0005201~extracellular matrix structural constituent | 40 | 40/1227 | 2.36E-12 | 2.47E-10 | 3.4585827 |
| GO_MF | GO:0004674~protein serine/threonine kinase activity | 73 | 73/1227 | 4.77E-12 | 4.51E-10 | 2.3543293 |
| GO_MF | GO:0004896~cytokine receptor activity | 28 | 28/1227 | 7.78E-11 | 6.04E-09 | 4.0682916 |
| GO_MF | GO:0038024~cargo receptor activity | 26 | 26/1227 | 8.84E-11 | 6.04E-09 | 4.3110216 |
| GO_MF | GO:0016504~peptidase activator activity | 17 | 17/1227 | 1.81E-10 | 1.14E-08 | 6.3050873 |
| GO_MF | GO:0019207~kinase regulator activity | 42 | 42/1227 | 3.87E-10 | 2.28E-08 | 2.8734778 |
| GO_MF | GO:0002020~protease binding | 31 | 31/1227 | 7.95E-10 | 4.41E-08 | 3.4402005 |
| GO_MF | GO:0008201~heparin binding | 36 | 36/1227 | 2.00E-09 | 1.05E-07 | 3.0022135 |
| GO_MF | GO:0005518~collagen binding | 21 | 21/1227 | 3.46E-09 | 1.72E-07 | 4.417436 |
| GO_MF | GO:0001618~virus receptor activity | 22 | 22/1227 | 4.48E-09 | 2.01E-07 | 4.1900262 |
| GO_MF | GO:0104005~hijacked molecular function | 22 | 22/1227 | 4.48E-09 | 2.01E-07 | 4.1900262 |
| GO_MF | GO:1901681~sulfur compound binding | 45 | 45/1227 | 5.95E-09 | 2.55E-07 | 2.5368704 |
| GO_MF | GO:0004298~threonine-type endopeptidase activity | 11 | 11/1227 | 2.03E-08 | 7.37E-07 | 7.7515485 |
| GO_MF | GO:0070003~threonine-type peptidase activity | 11 | 11/1227 | 2.03E-08 | 7.37E-07 | 7.7515485 |
| GO_MF | GO:0019199~transmembrane receptor protein kinase activity | 20 | 20/1227 | 4.19E-07 | 1.28E-05 | 3.5680315 |
| GO_MF | GO:0043028~cysteine-type endopeptidase regulator activity involved in apoptotic process | 14 | 14/1227 | 6.11E-07 | 1.69E-05 | 4.6979082 |
| GO_MF | GO:0042056~chemoattractant activity | 13 | 13/1227 | 1.54E-06 | 3.83E-05 | 4.6979082 |
| GO_MF | GO:0060090~molecular adaptor activity | 38 | 38/1227 | 1.64E-06 | 3.97E-05 | 2.2693285 |
| GO_MF | GO:0046332~SMAD binding | 19 | 19/1227 | 2.31E-06 | 5.19E-05 | 3.3472596 |
| GO_MF | GO:0042277~peptide binding | 42 | 42/1227 | 5.44E-06 | 0.0001168 | 2.0697078 |
| GO_MF | GO:0044325~ion channel binding | 24 | 24/1227 | 5.68E-06 | 0.0001191 | 2.7278177 |
| GO_MF | GO:0043531~ADP binding | 12 | 12/1227 | 7.39E-06 | 0.0001516 | 4.4506499 |
| GO_MF | GO:0005543~phospholipid binding | 55 | 55/1227 | 9.86E-06 | 0.0001981 | 1.8281954 |
| GO_MF | GO:0005089~Rho guanyl-nucleotide exchange factor activity | 18 | 18/1227 | 1.37E-05 | 0.0002596 | 3.0937444 |
| GO_MF | GO:0000217~DNA secondary structure binding | 9 | 9/1227 | 2.18E-05 | 0.000397 | 5.2851467 |
| GO_MF | GO:1990782~protein tyrosine kinase binding | 19 | 19/1227 | 2.37E-05 | 0.0004138 | 2.8793631 |
| GO_MF | GO:0030169~low-density lipoprotein particle binding | 7 | 7/1227 | 5.76E-05 | 0.0008112 | 6.1660045 |
| GO_MF | GO:0050700~CARD domain binding | 7 | 7/1227 | 5.76E-05 | 0.0008112 | 6.1660045 |
| GO_MF | GO:0017048~Rho GTPase binding | 28 | 28/1227 | 6.28E-05 | 0.0008596 | 2.204605 |
| GO_MF | GO:0000287~magnesium ion binding | 31 | 31/1227 | 0.0001149 | 0.0014291 | 2.0416143 |
| GO_MF | GO:0050840~extracellular matrix binding | 13 | 13/1227 | 0.0001156 | 0.0014291 | 3.2717575 |
| GO_MF | GO:0016853~isomerase activity | 25 | 25/1227 | 0.0001261 | 0.001497 | 2.2300197 |
| GO_MF | GO:0003684~damaged DNA binding | 14 | 14/1227 | 0.0001269 | 0.001497 | 3.0830022 |
| GO_MF | GO:0008022~protein C-terminus binding | 28 | 28/1227 | 0.0001365 | 0.0015711 | 2.1102903 |
| GO_MF | GO:0043394~proteoglycan binding | 10 | 10/1227 | 0.000144 | 0.0016188 | 3.9149235 |
| GO_MF | GO:0070888~E-box binding | 12 | 12/1227 | 0.0001497 | 0.0016631 | 3.3824939 |
| GO_MF | GO:0008200~ion channel inhibitor activity | 10 | 10/1227 | 0.000185 | 0.0020302 | 3.8091147 |
| GO_MF | GO:0042288~MHC class I protein binding | 7 | 7/1227 | 0.0002099 | 0.0022424 | 5.1924248 |
| GO_MF | GO:0051087~chaperone binding | 18 | 18/1227 | 0.0002429 | 0.0024654 | 2.5117529 |
| GO_MF | GO:0008329~signaling pattern recognition receptor activity | 7 | 7/1227 | 0.0003032 | 0.003045 | 4.9328036 |
| GO_MF | GO:0005072~transforming growth factor beta receptor, cytoplasmic mediator activity | 5 | 5/1227 | 0.0003324 | 0.0032016 | 7.0468623 |
| GO_MF | GO:0045294~alpha-catenin binding | 5 | 5/1227 | 0.0003324 | 0.0032016 | 7.0468623 |
| GO_MF | GO:0051015~actin filament binding | 28 | 28/1227 | 0.0003606 | 0.0033744 | 1.993052 |
| GO_MF | GO:0045499~chemorepellent activity | 8 | 8/1227 | 0.000412 | 0.003776 | 4.1759184 |
| GO_MF | GO:0038187~pattern recognition receptor activity | 7 | 7/1227 | 0.0004271 | 0.0038769 | 4.6979082 |
| GO_MF | GO:0005504~fatty acid binding | 9 | 9/1227 | 0.0004585 | 0.0040879 | 3.7306918 |
| GO_MF | GO:0051879~Hsp90 protein binding | 10 | 10/1227 | 0.000459 | 0.0040879 | 3.4374938 |
| GO_MF | GO:0001786~phosphatidylserine binding | 12 | 12/1227 | 0.0005503 | 0.0048349 | 2.9670999 |
| GO_MF | GO:0001228~DNA-binding transcription activator activity, RNA polymerase II-specific | 50 | 50/1227 | 0.000554 | 0.0048349 | 1.6125543 |
| GO_MF | GO:0001530~lipopolysaccharide binding | 9 | 9/1227 | 0.0005788 | 0.0048349 | 3.6241006 |
| GO_MF | GO:0015026~coreceptor activity | 10 | 10/1227 | 0.0008364 | 0.0065253 | 3.2031192 |
| GO_MF | GO:0045295~gamma-catenin binding | 5 | 5/1227 | 0.0009263 | 0.0071092 | 5.8723852 |
| GO_MF | GO:0048306~calcium-dependent protein binding | 12 | 12/1227 | 0.0010424 | 0.0078722 | 2.772536 |
| GO_MF | GO:0072341~modified amino acid binding | 15 | 15/1227 | 0.0011093 | 0.0082103 | 2.4299525 |
| KEGG | hsa00565~Ether lipid metabolism | 10 | 10/82 | 7.50E-05 | 0.0042008 | 4.1911399 |
| KEGG | hsa00360~Phenylalanine metabolism | 4 | 4/82 | 0.0076492 | 0.1525801 | 4.8321377 |
| KEGG | hsa00600~Sphingolipid metabolism | 7 | 7/82 | 0.0081739 | 0.1525801 | 2.9337979 |
| KEGG | hsa00380~Tryptophan metabolism | 6 | 6/82 | 0.0142182 | 0.199055 | 2.9337979 |
| KEGG | hsa00564~Glycerophospholipid metabolism | 10 | 10/82 | 0.017867 | 0.2001103 | 2.0955699 |
| KEGG | hsa00561~Glycerolipid metabolism | 7 | 7/82 | 0.0258799 | 0.2222205 | 2.3566573 |
| KEGG | hsa00350~Tyrosine metabolism | 5 | 5/82 | 0.0277776 | 0.2222205 | 2.8523035 |
| KEGG | hsa00230~Purine metabolism | 11 | 11/82 | 0.0418159 | 0.2927115 | 1.7648628 |
| KEGG | hsa00410~beta-Alanine metabolism | 4 | 4/82 | 0.054509 | 0.3082696 | 2.7382114 |
| KEGG | hsa00240~Pyrimidine metabolism | 6 | 6/82 | 0.0550481 | 0.3082696 | 2.1617458 |
| KEGG | hsa00983~Drug metabolism - other enzymes | 7 | 7/82 | 0.0851459 | 0.4104749 | 1.8196974 |
| KEGG | hsa00340~Histidine metabolism | 3 | 3/82 | 0.0879589 | 0.4104749 | 2.8004435 |
| KEGG | hsa01200~Carbon metabolism | 9 | 9/82 | 0.1144371 | 0.4929598 | 1.5663497 |
| KEGG | hsa00601~Glycosphingolipid biosynthesis - lacto and neolacto series | 3 | 3/82 | 0.140818 | 0.5632721 | 2.2818428 |
| KEGG | hsa00030~Pentose phosphate pathway | 3 | 3/82 | 0.176605 | 0.5707027 | 2.0536585 |
| KEGG | hsa00590~Arachidonic acid metabolism | 5 | 5/82 | 0.1803736 | 0.5707027 | 1.6561762 |
| KEGG | hsa00310~Lysine degradation | 5 | 5/82 | 0.1888642 | 0.5707027 | 1.6298877 |
| KEGG | hsa00520~Amino sugar and nucleotide sugar metabolism | 4 | 4/82 | 0.1924138 | 0.5707027 | 1.7477945 |
| KEGG | hsa00280~Valine, leucine and isoleucine degradation | 4 | 4/82 | 0.2026122 | 0.5707027 | 1.7113821 |
| KEGG | hsa00051~Fructose and mannose metabolism | 3 | 3/82 | 0.2146196 | 0.5707027 | 1.8669623 |
| KEGG | hsa00330~Arginine and proline metabolism | 4 | 4/82 | 0.2234807 | 0.5707027 | 1.6429268 |
| KEGG | hsa00010~Glycolysis / Gluconeogenesis | 5 | 5/82 | 0.2242046 | 0.5707027 | 1.532581 |
| KEGG | hsa00532~Glycosaminoglycan biosynthesis - chondroitin sulfate / dermatan sulfate | 2 | 2/82 | 0.2356986 | 0.5738748 | 2.1617458 |
| KEGG | hsa00770~Pantothenate and CoA biosynthesis | 2 | 2/82 | 0.2726104 | 0.636091 | 1.9558653 |
| KEGG | hsa00220~Arginine biosynthesis | 2 | 2/82 | 0.2910756 | 0.6419029 | 1.8669623 |
| KEGG | hsa00260~Glycine, serine and threonine metabolism | 3 | 3/82 | 0.308417 | 0.6419029 | 1.5402439 |
| KEGG | hsa00515~Mannose type O-glycan biosynthesis | 2 | 2/82 | 0.3094889 | 0.6419029 | 1.78579 |
| KEGG | hsa00592~alpha-Linolenic acid metabolism | 2 | 2/82 | 0.3460103 | 0.6920207 | 1.6429268 |
| KEGG | hsa00982~Drug metabolism - cytochrome P450 | 4 | 4/82 | 0.4362575 | 0.8038431 | 1.1905267 |
| KEGG | hsa00270~Cysteine and methionine metabolism | 3 | 3/82 | 0.4436771 | 0.8038431 | 1.2321951 |
| KEGG | hsa00640~Propanoate metabolism | 2 | 2/82 | 0.5000216 | 0.8038431 | 1.2080344 |
| KEGG | hsa00760~Nicotinate and nicotinamide metabolism | 2 | 2/82 | 0.5000216 | 0.8038431 | 1.2080344 |
| KEGG | hsa00533~Glycosaminoglycan biosynthesis - keratan sulfate | 1 | 1/82 | 0.5042325 | 0.8038431 | 1.466899 |
| KEGG | hsa00480~Glutathione metabolism | 3 | 3/82 | 0.5078233 | 0.8038431 | 1.1201774 |
| KEGG | hsa00500~Starch and sucrose metabolism | 2 | 2/82 | 0.5157273 | 0.8038431 | 1.1735192 |
| KEGG | hsa00603~Glycosphingolipid biosynthesis - globo and isoglobo series | 1 | 1/82 | 0.5285756 | 0.8038431 | 1.3691057 |
| KEGG | hsa00250~Alanine, aspartate and glutamate metabolism | 2 | 2/82 | 0.5311106 | 0.8038431 | 1.1409214 |
| KEGG | hsa00910~Nitrogen metabolism | 1 | 1/82 | 0.5737741 | 0.8260302 | 1.2080344 |
| KEGG | hsa00620~Pyruvate metabolism | 2 | 2/82 | 0.575271 | 0.8260302 | 1.0531582 |
| KEGG | hsa00100~Steroid biosynthesis | 1 | 1/82 | 0.6336634 | 0.8677171 | 1.0268293 |
| KEGG | hsa00071~Fatty acid degradation | 2 | 2/82 | 0.6420864 | 0.8677171 | 0.9334812 |
| KEGG | hsa00514~Other types of O-glycan biosynthesis | 2 | 2/82 | 0.6781137 | 0.8677171 | 0.8738973 |
| KEGG | hsa00534~Glycosaminoglycan biosynthesis - heparan sulfate / heparin | 1 | 1/82 | 0.7007655 | 0.8677171 | 0.8556911 |
| KEGG | hsa00562~Inositol phosphate metabolism | 3 | 3/82 | 0.7022799 | 0.8677171 | 0.8439693 |
| KEGG | hsa00510~N-Glycan biosynthesis | 2 | 2/82 | 0.7111812 | 0.8677171 | 0.8214634 |
| KEGG | hsa00062~Fatty acid elongation | 1 | 1/82 | 0.7429796 | 0.8677171 | 0.7606143 |
| KEGG | hsa01040~Biosynthesis of unsaturated fatty acids | 1 | 1/82 | 0.7429796 | 0.8677171 | 0.7606143 |
| KEGG | hsa00591~Linoleic acid metabolism | 1 | 1/82 | 0.7677958 | 0.8677171 | 0.7081581 |
| KEGG | hsa00053~Ascorbate and aldarate metabolism | 1 | 1/82 | 0.7793008 | 0.8677171 | 0.6845528 |
| KEGG | hsa00630~Glyoxylate and dicarboxylate metabolism | 1 | 1/82 | 0.7793008 | 0.8677171 | 0.6845528 |
| KEGG | hsa00052~Galactose metabolism | 1 | 1/82 | 0.7902424 | 0.8677171 | 0.6624705 |
| KEGG | hsa00513~Various types of N-glycan biosynthesis | 1 | 1/82 | 0.8605048 | 0.9266975 | 0.5265791 |
| KEGG | hsa00970~Aminoacyl-tRNA biosynthesis | 1 | 1/82 | 0.8920094 | 0.9425005 | 0.4667406 |
| KEGG | hsa00190~Oxidative phosphorylation | 3 | 3/82 | 0.9636714 | 0.9783103 | 0.4667406 |
| KEGG | hsa00830~Retinol metabolism | 1 | 1/82 | 0.9670793 | 0.9783103 | 0.3065162 |
| KEGG | hsa00980~Metabolism of xenobiotics by cytochrome P450 | 1 | 1/82 | 0.9783103 | 0.9783103 | 0.2738211 |
