# Supplementary figures and images for: Construction and Validation of an Immune-Related Gene Prognostic Index for Esophageal Squamous Cell Carcinoma
Source: Biomed Res Int. 2021 Oct 21;2021:7430315. doi: 10.1155/2021/7430315 (PMC8553461; doi:10.1155/2021/7430315)

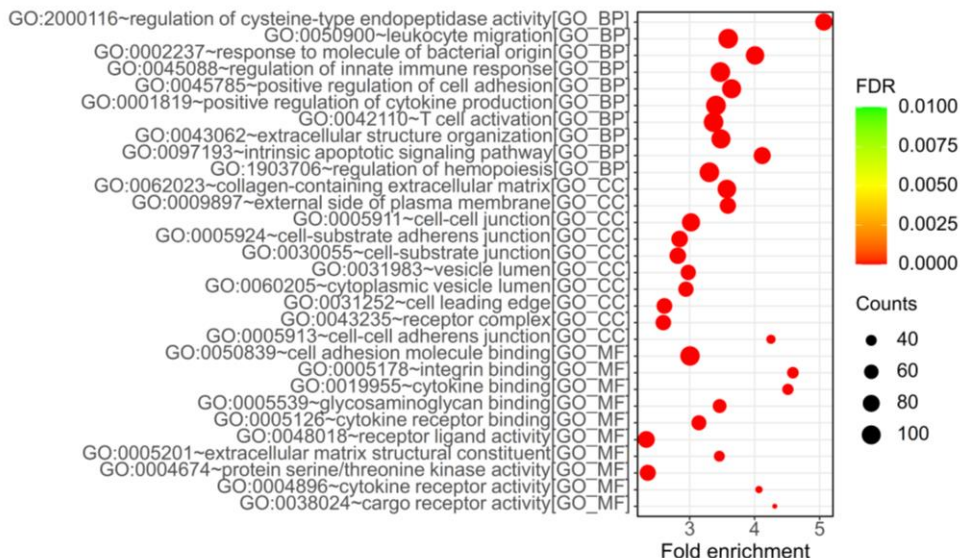

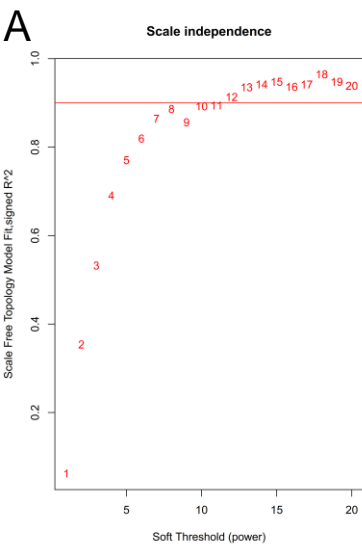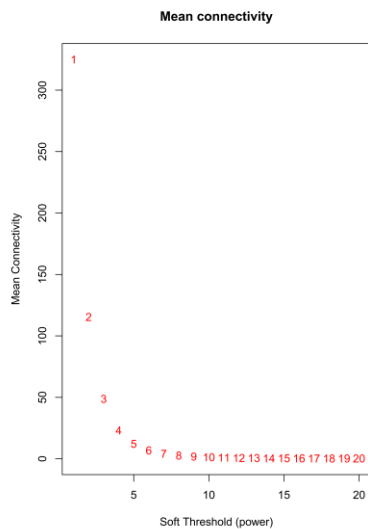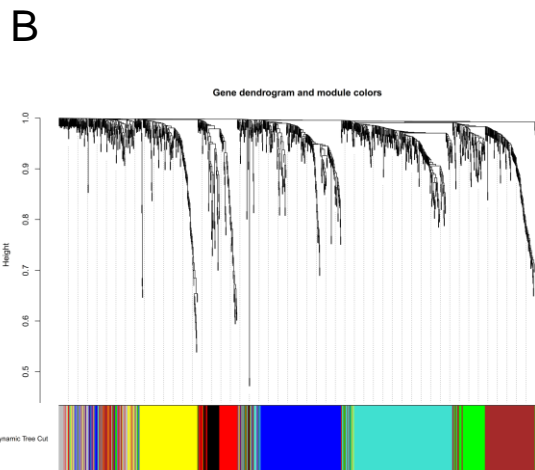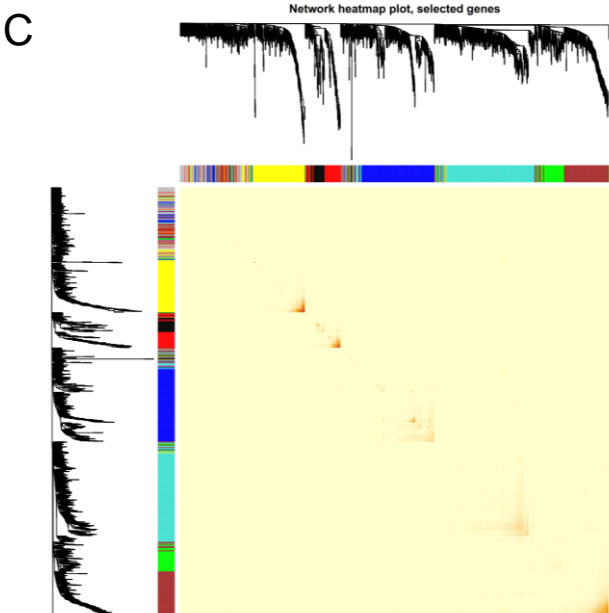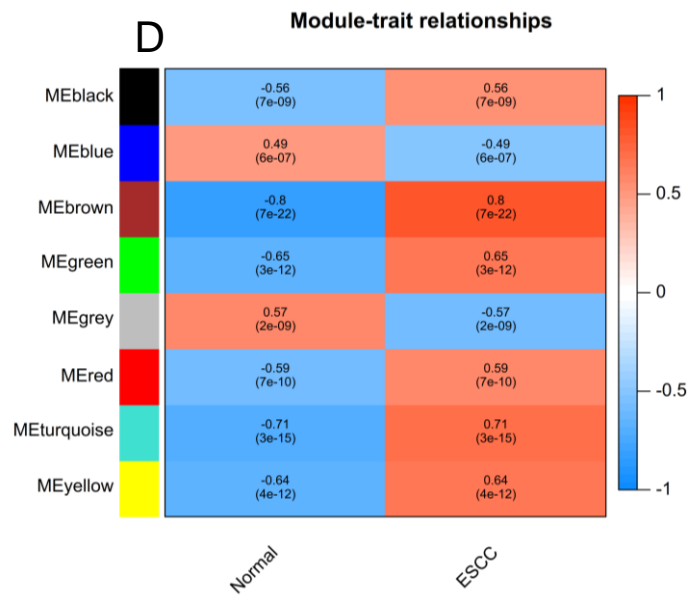

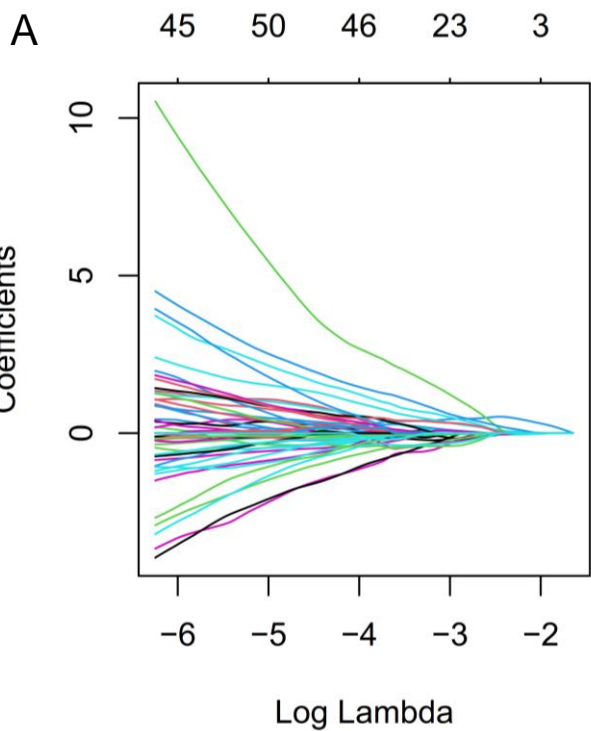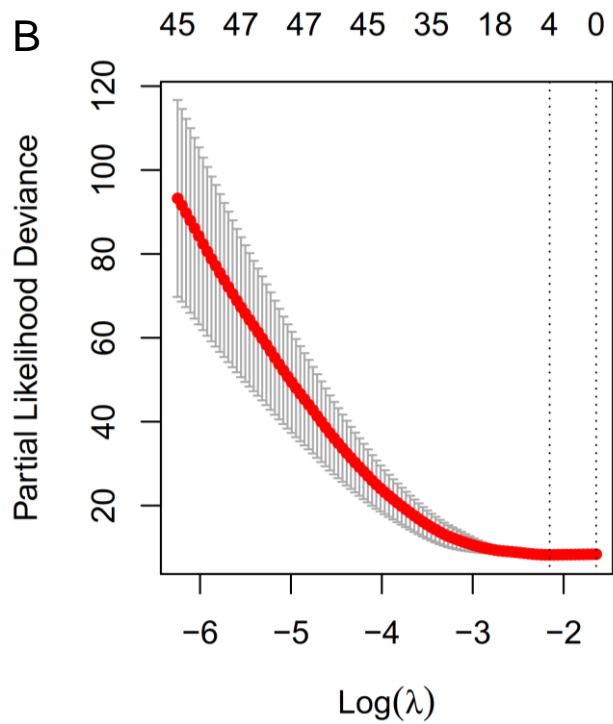

Supplement: Supplementary Materials — Supplementary Figure S1: Gene ontology (GO) enrichment analysis of differentially expressed immune-related genes in the TCGA ESCC cohort. Supplementary Figure S2: WGCNA method used to identify the hub genes. (A) Determination of soft threshold in WGCNA. (B) WGCNA analysis of differentially expressed immune-related genes in ESCC and the gene modules. (C) Gene expression clustering tree and coexpression topological heat map. (D) Relationship between the gene modules and features of ESCC. Supplementary Figure S3: differentially expressed immune-related genes analyzed using lasso Cox regression to determine genes included in the prognostic model. (A) Lasso Cox regression fitting process. (B) Tenfold crossvalidation was used to determine the λ values. The lambda value in lasso Cox reflects the degree of regularization. The larger the lambda value, the fewer effective variables are screened. Tenfold crossvalidation was used to determine the lambda values; the minimum crossvalidation error is set to the best lambda value. Supplementary Table S1: primers used for qPCR. Supplementary Table S2: GO terms and KEGG pathway enrichment analyses of differentially expressed immune-related genes of ESCC. Supplementary Table S3: GSEA enrichment analysis between IRGPI subgroups. [file 7430315.f1.zip › Supplementary_Figures.pdf]
